# A Suite of Pea (*Pisum sativum* L.) Near-Isolines: Genetic Resources and Molecular Tools to Breed for Seed Carbohydrate and Protein Quality in Legumes

**DOI:** 10.3390/ijms26062612

**Published:** 2025-03-14

**Authors:** Tracey Rayner, Julia E. A. Mundy, Lorelei J. Bilham, Carol Moreau, David M. Lawson, Claire Domoney, Trevor L. Wang

**Affiliations:** 1 John Innes Centre, Norwich NR4 7UH, UK; tracey.rayner@jic.ac.uk (T.R.); julia.mundy@jic.ac.uk (J.E.A.M.); l.bilham@uea.ac.uk (L.J.B.); carolj.moreau@gmail.com (C.M.); david.lawson@jic.ac.uk (D.M.L.); trevor.wang@jic.ac.uk (T.L.W.); 2 School of Biological Sciences, University of East Anglia, Norwich NR4 7TJ, UK; 3 Paleogenomics Laboratory, Génétique Diversité Ecophysiologie des Céréales, l’Institut National de Recherche pour l’Agriculture, l’Alimentation et l’Environnement, 5 Chemin de Beaulieu, 63000 Clermont-Ferrand, France

**Keywords:** amylose, amylopectin, breeding, legumes, modelling, mutant, pea, *Pisum sativum*, protein, starch

## Abstract

In recent years there has been a resurgent interest in plant products as substitutes for animal-derived food products, in which legumes, including peas, feature highly. Here, we report on a set of *Pisum sativum* L. (pea) near-isolines, comprising 24 unique mutants at five loci, where the impact of the mutations on the corresponding enzymes of the starch pathway confers a wrinkled-seeded phenotype. Together with a set of round-seeded mutants impacted at a sixth locus, all 27 mutants show variation for starch composition and protein content. The mutations have been mapped onto three-dimensional protein models to examine potential effects on the corresponding enzyme structures and their activities, and to guide targeted mutagenesis. The mutant lines represent a unique suite of alleles for rapid introduction into elite pea varieties to create new materials for the food and feed markets and industrial applications.

## 1. Introduction

The rising interest in plant-based foods and ingredients as alternatives to animal-derived products has led to an increase in the production of plant protein that is estimated to grow in value by over 40% in the next five years [1]. Pea constitutes one of the four major plant-based proteins globally [2] and is eminently suitable for supplying this market, especially in Europe and Canada [3,4]. Moreover, pea starch is a useful byproduct of protein extraction for both food and non-food use. It also has unique properties [5,6,7] and is classed as a non-conventional starch type [8]. Most current uses, however, other than frozen peas (vining pea) for human nutrition, rely on just one genotype of pea—the combining pea—grown for its mature seeds, which are mostly round (smooth)-seeded varieties and have smooth starch grains [9,10]. Very little investigation has been undertaken to date on the full range of pea germplasm that can be used either for nutrition or for protein and starch extraction for use in industrial applications.

Altering the starch and protein composition of the seed can have a profound effect on the nutritional quality of the crop, as well as on the processing of the seed for its components [11,12,13]. Six loci are known to affect the starch composition of the seed [5], and several are known to have pleiotropic effects on the protein components [14]. These are the five *rugosus* (meaning wrinkled) loci—*r*, *rb*, *rug3*, *rug4*, and *rug5*—where the mature dry seed is wrinkled, and the *lam* (*low amylose*) locus, which has smooth (or round) seeds. The *rugosus* and *lam* loci encode enzymes of the starch biosynthetic pathway, which have been reviewed recently in the pea [15]. The naturally occurring *r* locus mutant used by Mendel in his seminal experiments [16] possesses an insertion in the gene encoding starch branching enzyme I (*sbe1*; [17]) that causes a decrease in the starch content of the seed with a concomitant increase in the amylose content of the starch, resulting in a compound starch grain. Another naturally occurring mutant, *rb*, first described by Kooistra [18], affects the activity of ADP glucose pyrophosphorylase (AGPase; [19]), impacting the large subunit of this enzyme (AGPL). Mutants at the *rb* locus have lower starch in their seeds than the wild type, as well as less amylose. Their starch grains are, however, smooth like those of the wild type. Mutants at the *rug3* locus show the most severe wrinkled-seeded phenotype. This locus encodes plastidial phosphoglucomutase, the enzyme that is the first step for starch synthesis in the plastid (pPGM; [20]). The absence of this enzyme prevents the production of ADP-glucose, and hence the seeds contain no starch. In weaker alleles, the starch grains are smooth but disc-shaped [21]. The *rug4* locus encodes a sucrose synthase providing substrate to the plastid. It has a mild effect on the starch content of the seed, but a more profound effect on the assimilation of nitrogen via the nodule [22] as the encoded isoform, SUS1, is highly expressed in the nodule [23]. Mutants at the *rug5* locus that encodes starch synthase 2 (SS2) possess highly compound irregular starch granules as their mutations cause compensatory changes in other enzymes of the starch biosynthetic pathway. The consequence is a profound alteration to the amylopectin structure of the starch [24]. By contrast to the *rugosus* mutants, those at the *lam* locus, which encodes granule-bound starch synthase 1 (GBSS1), have round seeds indistinguishable from the wild type. These mutants were isolated on the basis of the iodine staining of their starch granules [25]; they do not have significantly less starch in their seeds than the wild type (hence the seeds are round/smooth), but the mutations lower the amylose content of the starch profoundly [5]. This creates the appearance of a pale red-brown halo around iodine-stained *lam* granules when viewed under a microscope [25].

One consequence of mutations leading to a lowering of the starch level in the seed is that the proportion of protein available is increased, which should aid in its separation during purification. This may be a valuable characteristic given the processes required for protein extraction [26,27]. There are also other loci that are known to influence protein quality directly (e.g., [13]), and numerous quantitative trait loci have been identified that affect protein composition [28,29].

Here we provide molecular information on unique mutants at five loci in peas, which—together with information from a sixth locus reported previously [30] and relevant gene sequences derived from a high-quality genome assembly for the pea accession, JI2822 [31]—provide a comprehensive dataset for researchers and pea breeders. The information provided supports the rapid introduction of unique mutations into elite field varieties to deliver pea material with a range of properties. In addition, it provides insights into the effects of the mutations on the structures of their respective enzymes.

## 2. Results

### 2.1. Seed Phenotypes

The original suite of mutants, characterised on the basis of seed shape, starch content and genetic complementation, consisted of 32 lines. The original line number designations for the *rugosus* wrinkled-seeded mutants were assigned by Wang and Hedley [32], and the loci by Wang et al. [5]. The low amylose mutants were isolated later through a separate screen [25], five lines of which were purified and designated alleles at the *lam* locus [5].

**Figure 1:**
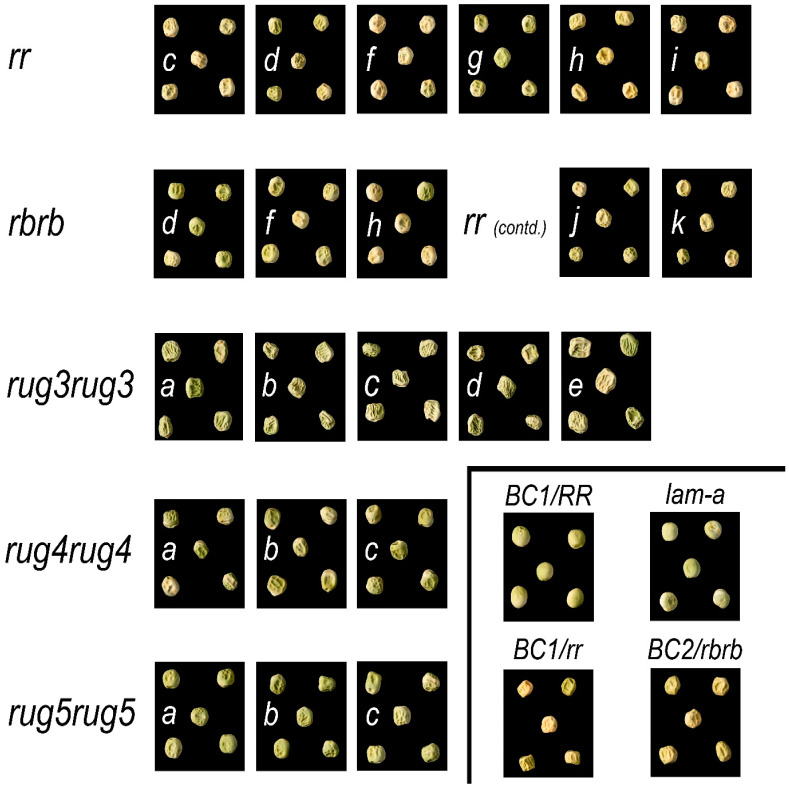
Seed phenotypes of pea mutants affected in starch biosynthesis. Five representative seeds are shown for each line. The lettering on each image of a *rugosus* mutant represents the allele suffix, i.e., for *rug3*, the *a* denotes the *rug3-a* allele. Only mutants identified as unique by sequencing are shown. For reference, seeds of the BC1/RR line are 6 mm in diameter.

Following the identification of each mutation (see Section 2.2), the suite of induced mutants was reduced to 25 unique alleles, 7 of which have been reported elsewhere [30]. The seed phenotypes of the mutants are shown in Figure 1. For completeness, images of the *rugosus* (*r*) locus alleles that have been reported previously [30] have also been included, as well as the naturally occurring mutant at the *rugosus-b* (*rb*) locus. For reference, the round-seeded parental line (BC1/RR; JI3316) used for the mutagenesis is included in Figure 1, together with the backcross lines generated for *r* and *rb* (JI3317 and JI3319, respectively). All of the lines affected in their starch content are wrinkled-seeded, with the exception of the *lam* mutants, which are round-seeded and identical to the parental line because the primary effect of the *lam* locus is to alter starch composition rather than amount. For this reason, seeds from only one *lam* mutant are shown (Figure 1). As reported previously [21], *rug3* has the most severe effect on the wrinkling of the seed, often leading to the collapse of the seed, as can be seen in Figure 1.

### 2.2. Unique Alleles

Each mutant allele of the original suite of 32 was sequenced following primer design based on the NCBI/GenBank sequences for the respective wild-type gene or mRNA from pea and, where required, the orthologue in *Medicago truncatula* (Mt) for prediction of intron/exon structure. For the *r* mutants reported previously, mutations were sought first of all through cDNA sequencing and then validated through genomic sequencing; the latter provided an understanding of the basis of multiple transcripts for some mutants [30]. The same approach was taken here for *rb* mutations, whereas for all other mutants, genomic amplification and sequencing were adopted.

Table 1 describes the nature of the mutations at each of the six pea loci and brings together (with corrections where necessary) all the relevant data, including that from previous articles where the mutations in single alleles were identified for each of the *rug3*, *rug4,* and *rug5* loci and used to characterise the lesion in each mutant [20,22,24]. The set of mutations at the *r* locus was reported previously [30] and is included here with the addition of original isolation numbers for completeness.

The original isolation numbers bear reference to the mutagen used [34]. Both EMS and MNU induced mutations, although EMS was more effective, with 84% of the alleles induced by EMS. The mutagens caused mainly (84%) G to A transitions, with only four C to T transitions (16%); the two naturally occurring mutations were either due to an insertion or a deletion (*r* and *rb*, respectively).

**Figure 2:**
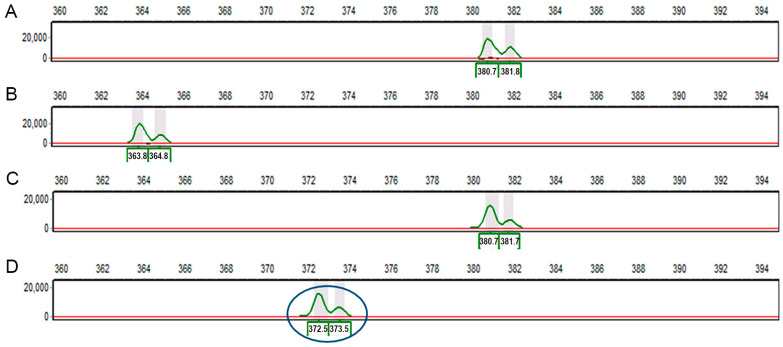
PCR amplification of the region of the *AGPL1* gene which covers the region where the 9 bp deletion in *rb* mutants is located, showing three size variants, two of which are wild type—(**A**) JI0281; (**B**) JI1194; (**C**) JI0015—and one of which is *rb* (**D**) JI0399 (oval shape). A variant intron in a wild-type *AGPL1* gene (17 bp deletion in JI1194) is revealed using this assay, based on using primers PsAGP1L_F1 and PsAGP1L_R8, where the former includes the fluorescently labelled tail. Amplicon size determination relative to fluorescent markers followed chromatographic analysis. The shoulder on the amplicon peak in each case (green trace) reflects the tendency of Taq polymerase to add a non-templated residue (**A**) to newly synthesised strands of DNA; the double peaks are highlighted in grey in every case.

The complete sequences of the five genes and cDNAs studied in this paper, and the primers that can be used to identify each mutation for rapid screening within crossing or breeding programmes, are provided in Supplementary Figure S1 and Supplementary Table S1, respectively. For the naturally occurring *rb* mutation, a deletion mutant, it should be noted that, as there is natural variation in the length of the intron adjacent to the deletion, estimates of amplicon size alone may not be sufficient to identify lines carrying the mutation among progeny lines, depending on the genotypes being used for crossing. Figure 2 shows three allelic variants for the *AGPL* F1–R8 amplicon size distinguished using fluorescent primers; one of the variants (354 bp) reflects the presence of the *rb* mutation (9 bp deletion in JI0399, cv. Cennia), whereas the other two are wild-type alleles. The smaller amplicon obtained for the wrinkled-seeded line, JI1194, reflects a deletion of 17 bp from intron 2 of a wild-type *AGPL* gene (i.e., 346 bp, compared with 363 bp in JI0281 and JI0015 (Figure 2, showing amplicon sizes which include the M13 fluorescent tail). An intragenic recombination between the variant regions of JI1194 and JI0399 could generate a further size variant (337 bp).

### 2.3. Protein Structures

Structural models were computed from the corresponding protein sequences for each allele using AlphaFold2. The potential implications of mutations at a particular location are presented and discussed, based on related, empirically derived structural information from databases such as the Protein Data Bank (PDB). Further information on the predicted structures showing the quality of the models generated is given in Supplementary Figures S2–S6, respectively, for the proteins predicted from the five genes presented below. Note that amino acid sequence numbering in this section refers to the wild-type protein in every case (numbering in Table 1 is based on JI2822, a *rb* mutant with a three amino acid deletion).

#### 2.3.1. Mutants at the *rb* Locus (ADP-Glucose Pyrophosphorylase Large Subunit 1; Figure 3 and Figure 4)

**Figure 3:**
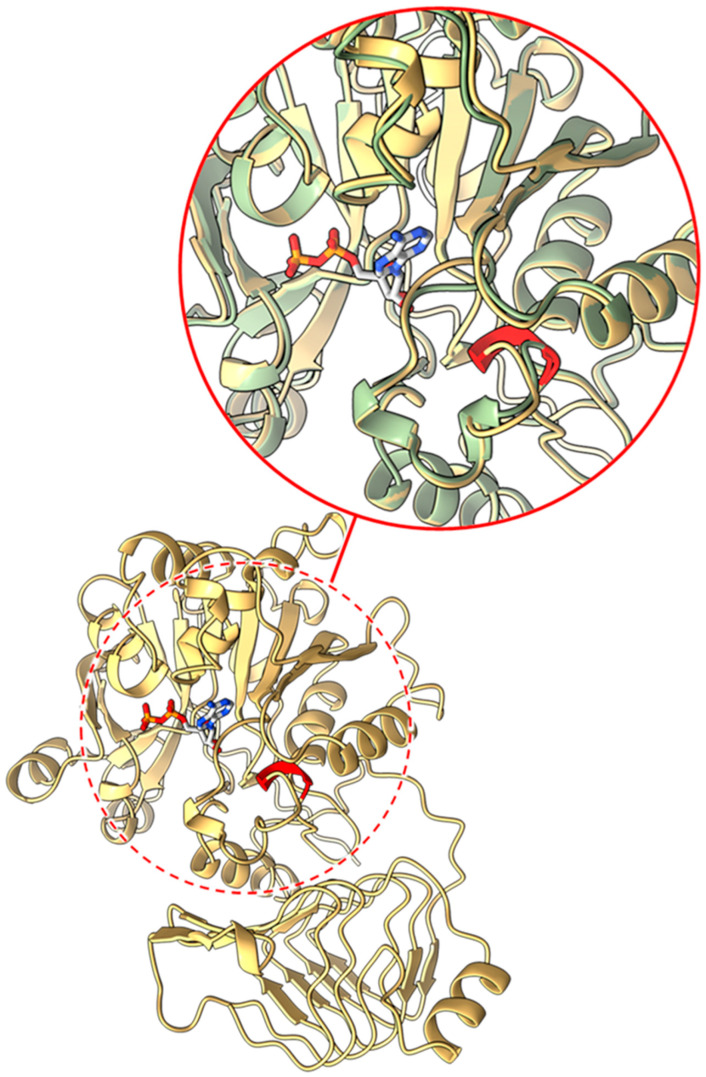
Model of the wild-type AGPL1 monomer is shown in the main panel. Within the circles, the deleted region is shown in solid red and the predicted position of ADP in the active site is shown in stick representation (blue, grey, red, orange). The inset (solid red circle, as expanded view of the region within the dashed circle) shows a close-up of this region overlaid with another AlphaFold model (pale green) computed from the *rb* sequence with wild-type residues (ATP/TPA; from position 99/100) removed to give an indication of the location and scale of the lost structure in the context of the folded protein.

*rb* (*naturally occurring allele*). The effect of this in-frame deletion of three amino acids from the AGPL1 protein is more challenging to predict using AlphaFold2, as it is not trained to predict the structural consequences of mutations. The deletion occurs around 100 amino acids into the sequence and may result in the rest of the structure being unable to fold properly (Figure 3). If the translated protein does fold, then there may be some local destabilisation to nearby α-helices, resulting in a loss of integrity at the active site or the sites of interactions for protein–protein interactions, which could affect protein function.

**Figure 4:**
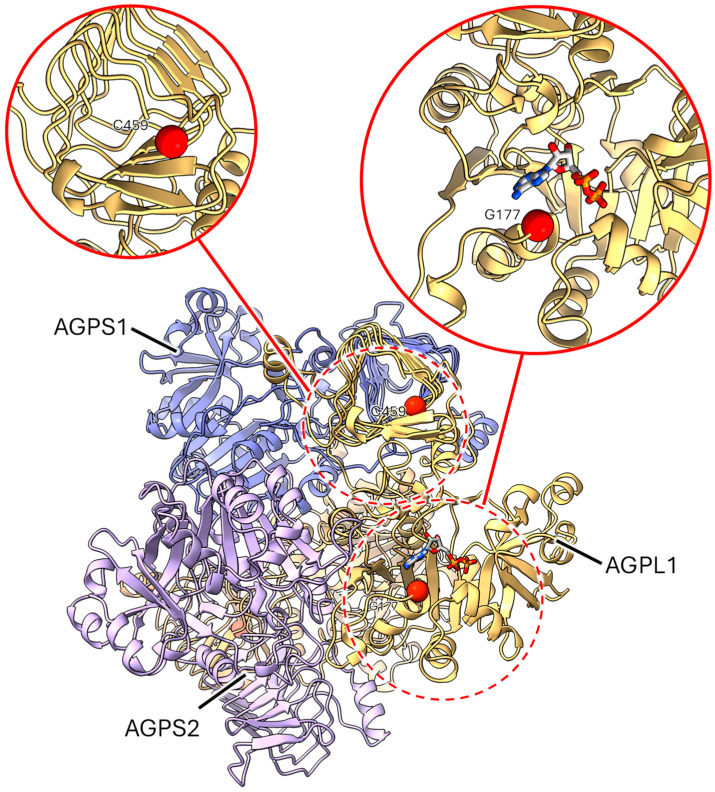
Location of pea ADP glucose pyrophosphorylase large subunit 1 mutations. The central panel shows the AGPS1 and AGPL1 A2B2 heterotetramer to give a view of the binding site on one of the AGPL1 subunits; expanded views (solid red line circles) of the site of two mutations (dashed red line circles) are shown above. The sites of the reported mutations are shown as solid red spheres and the ADP ligand (in stick representation, blue, grey, red, orange) taken from the related structure 1YP4. Mutation sites at G177 (*rb-h*) and C459 (*rb-d*) are highlighted to show the local structure in these regions.

*rb-d*. The side chain of Cys459 is predicted to point onto the core of the β-helix, which is crucial for tetramer formation (Figure 4). The C459Y substitution would introduce a much bulkier side chain here that could destabilise the secondary structure and in turn disrupt oligomerisation. Oligomer formation would be necessary for the stability and functioning of the enzyme.

*rb-f*. This mutation introduces a stop codon, resulting in the truncation of the protein after W307, which would result in partial loss of the catalytic domain and the complete absence of the β-helix domain. The resultant truncated polypeptide would be unlikely to have any enzymatic activity.

*rb-h*. From the ligands bound to a similar PDB accession 1YP4, it appears that the site of the *rb-h* mutation, resulting in a G177R substitution, is in close proximity to the nucleotide binding pocket in the active site. The substitution for a bulky positive side chain could introduce a steric clash with the adenine ring (Figure 4) and a concomitant reduction in the functionality of the enzyme.

The association of AGPL1 with ADP-glucose pyrophosphorylase small subunits 1 and 2 (AGPS1 and AGPS2 respectively) into heterotetramers was also modelled using AlphaFold2, with a composition of one molecule of both AGPS1 and AGPS2 bound with two molecules of AGPL1, yielding models which were largely similar to the arrangement shown in Figure 4. Mutations that could disrupt tetramerization are likely to negatively impact the formation of a functional and stable enzyme.

#### 2.3.2. Mutants at the *rug3* Locus (Plastidial Phosphoglucomutase; Figure 5)

*rug3-a* and *rug3-c*. The two glycine residues substituted in mutants *rug3-a* and *rug3-c*, G128D and G195S, respectively, are located in β-sheets that are within 10 Å of the likely active site based on superposition with PDB entry 6SNO, and as such are likely to impact substrate binding and catalysis.

**Figure 5:**
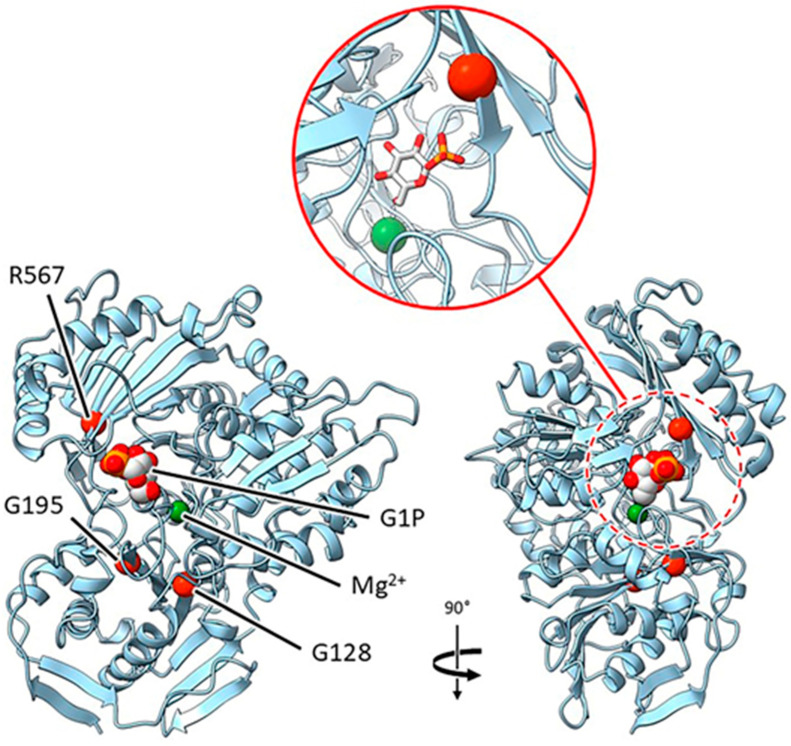
Location of pea plastidial phosphoglucomutase mutations. The main panel shows orthogonal views of the enzyme with the identified mutation sites represented by red spheres. The site in the view on the right (dashed red circle) has been expanded (solid red circle) to show the arrangement more clearly. Both glucose-1-phosphate (G1P) and the active site Mg^2+^ ion (green), were taken from PDB accession 6SNO. Corresponding alleles: G128—*rug3-a*; R567—*rug3-b*; G195—*rug3-c*.

*rug3-b*. The arginine lost by the R567C mutation in the *rug3-b* mutant is predicted to be an important active site residue involved in binding the phosphate group of glucose-1-phosphate. The loss of this interaction is likely to adversely affect the catalytic activity of the enzyme.

*rug3-d*. This mutation introduces a premature stop into the protein at R131, removing the majority of the protein structure. With a mutation of this severity, it is unlikely that a resultant truncated polypeptide would fold.

*rug3-e*. This deletion mutation results in the loss of a domain that represents one side of the active site pocket. The impact of this is the loss of key residues, including R567, which alone has severe phenotypic implications (see *rug3-b* and coordinating structures of the active site). In any case, this mutant would be unlikely to result in a folded protein.

#### 2.3.3. Mutants at the *rug4* Locus (Sucrose Synthase 1; Figure 6)

*rug4-a*. Comparison to similar enzymes indicated that the SUS1 protein would self- associate to form a homotetramer, exemplified in PDB accession 3S27. The L164 residue, substituted for F in the *rug4-a* mutation, is located in an α-helix close to an interface between subunits. Although the AlphaFold2 model suggests that this residue side chain would point inwards, rather than out towards an adjacent monomer, the change to a bulky aromatic side chain such as phenylalanine may alter the local structure or affect helix folding. This is predicted to interfere with the formation of this interface and destabilise the expected oligomer.

**Figure 6:**
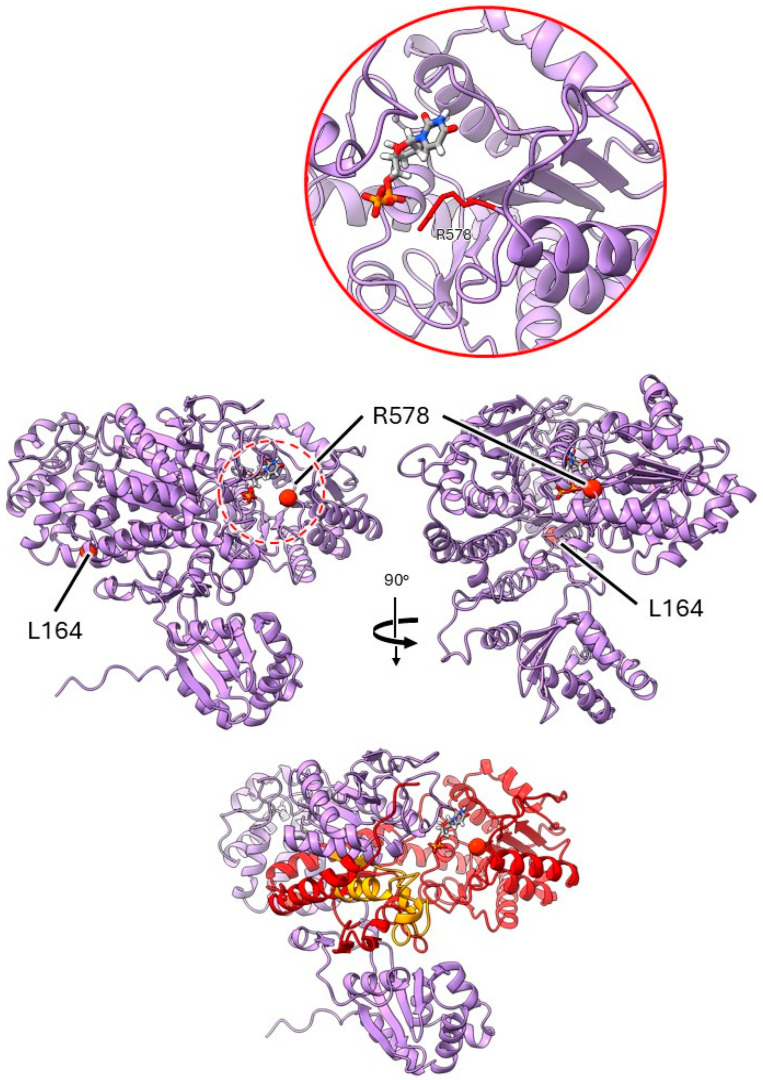
Location of pea sucrose synthase 1 mutations. Orthogonal views of the monomer are shown with the two mutation sites displayed as red spheres; the protein is predicted to form a homotetramer. The expanded view of the active site (solid red line circle) has added detail of the arginine side chain to demonstrate the proximity of the residue to the phosphate group of the ligand. The bottom plate indicates the region on the folded protein affected by the *rug4-c* mutation, with the sequence change shown in orange and the region lost to the premature stop codon indicated in red. Corresponding alleles: L164—*rug4-a*; R578—*rug4-b*.

*rug4-b*. There is evidence to suggest that the enzyme activity of SUS1 relies on hydrogen bonding between the phosphate group of the substrate and R578 in the active site [35]. Although the lysine substitution in the *rug4b* mutation could still play a role in substrate binding, the decrease in positive charge could have an impact on transition state stabilisation.

*rug4-c*. As with the amino acid deletion in the naturally occurring *rb* mutant protein, this cannot be reliably modelled using AlphaFold. However, we can predict that, while the *rug4-c* mutation resulting in 37 aberrant amino acids would adversely affect the phenotype, the loss of 338 amino acids C-terminal to the premature stop codon would undoubtedly be impactful, resulting in the loss of the majority of the active site.

#### 2.3.4. Mutants at the *rug5* Locus (Starch Synthase 2; Figure 7)

The first 200 residues of SS2 were poorly predicted by AlphaFold2, with this portion of the polymer exhibiting poor sequence coverage in the multiple sequence alignment and not aligning with any PDB accessions. Thus, it is not possible to comment directly on the function of this initial part of the protein, previously described as the ‘flexible arm’ [36]. This earlier study showed that the flexible N-terminal domain does not contribute to the enzyme’s catalytic activity. However, the core of the enzyme, where the mutations discussed herein are located, appeared to be well predicted by the algorithm, with good sequence coverage and confidence scores, and so further investigations focussed on this portion of the molecule, which has related structures present in the PDB.

**Figure 7:**
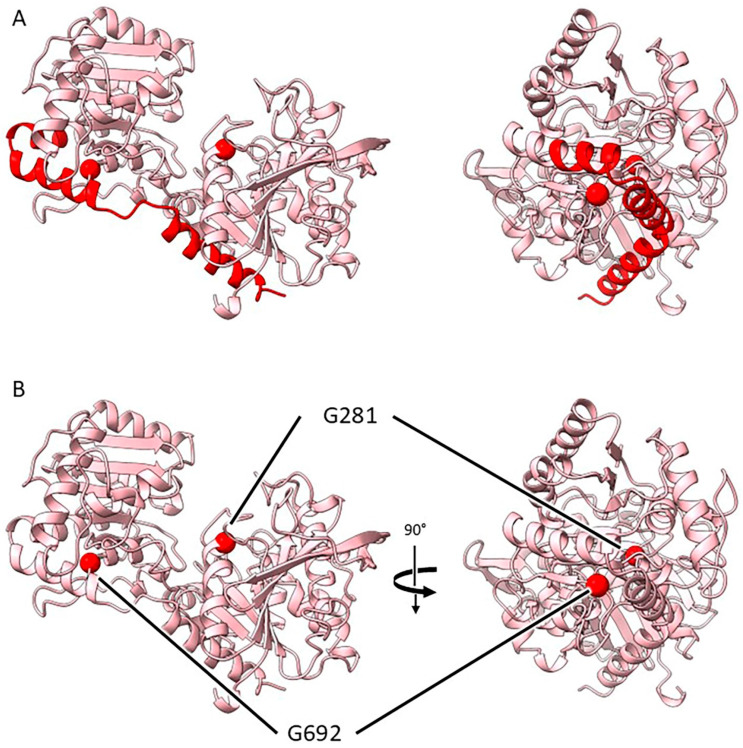
Location of the pea mutations in starch synthase 2 in two orthogonal views. (**A**) Deletion mutation *rug5-a* is represented by red colouring to indicate the loss of helical regions spanning both domains of the protein. (**B**) Sites of the point mutations in *rug5* are indicated using red spheres: G281—*rug5-b*; G692—*rug5-c*.

*rug5-a*. This mutation results in the deletion of three α-helices which bridge the two domains. The absence of these residues can be predicted to result in a protein that is either unable to fold properly or incapable of folding at all.

*rug5-b*. The substitution of the glycine (G281) for a much bulkier arginine at this location, the end of an α-helix close to the active site, can be predicted to cause disruption to the folding and local structure of this region.

*rug5-c*. The G692D substitution would most likely disrupt the local structure through the introduction of a side chain together with a negative charge into a tightly packed hydrophobic environment. Moreover, the wild-type glycine adopts backbone torsion angles that would be unfavourable for any other residue type, so any mutation here would likely result in a change in the protein backbone conformation. Although not directly adjacent to the active site, this local structural disruption is likely to have wider implications on protein folding and, in turn, activity.

#### 2.3.5. Mutants at the *lam* Locus (Granule-Bound Starch Synthase 1; Figure 8)

*lam-a*. For the *lam-a* mutation, the substituted residue is located on a connecting loop between a β-sheet and α-helix close to the active site region. Comparison to PDB accessions 3CX4 and 3VUF indicates that the glutamic acid (E85) is part of a hydrogen bonding network that stabilises a potentially mobile loop in the active site. Additionally, there is a carbohydrate moiety bound in PDB entry 3CX4 which is also in hydrogen bonding proximity to the corresponding glutamic acid, and so the substitution of this residue for a basic one (lysine) could cause disruption to these hydrogen bonds and destabilise the active site.

**Figure 8:**
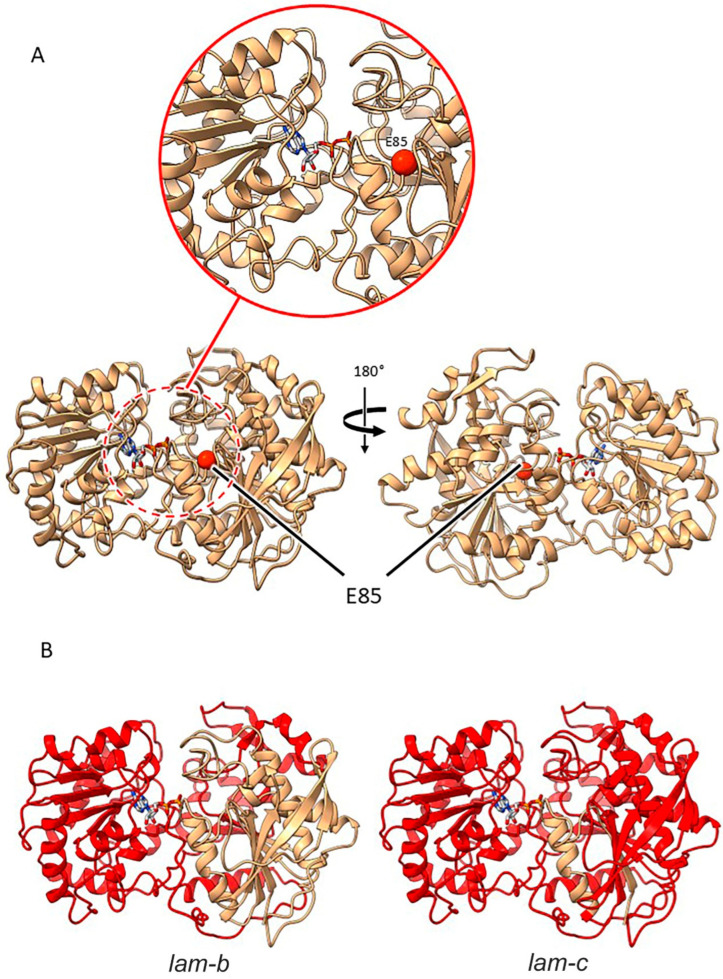
Location of pea mutations in granule bound starch synthase 1. (**A**) Site of the E85 mutation is displayed in the LAM protein as a red sphere to show the proximity to the active site (red dashed line circle), with a zoomed in section (red solid line circle) to focus on the active site. (**B**) Deletion mutations *lam-b* and *lam-c* indicate that the majority of the protein (represented by red colouring) is lost in both cases.

*lam-b*. The *lam-b* mutant is caused by a splice mutation and premature stop codon, resulting in a significantly truncated protein. The truncation in this case would remove a large portion of what, in comparison with other accessions in the PDB, includes the active site. The mutation predicts the introduction of a run of 12 aberrant amino acids before a premature stop codon, most likely preventing the proper folding of the encoded protein, which would certainly be catalytically inactive.

*lam-c*. This mis-splicing mutation, predicting a run of 11 aberrant amino acids and a premature stop in the protein, results in a truncation even more severe than *lam-b* and would give a non-functional protein.

## 3. Discussion

Two naturally occurring mutations have been described, where the impact on the shape of the seed is mediated via an effect on starch content of the cotyledons; these are *r* and *rb* [16,18]. Other naturally occurring mutants have been identified that affect the shape of the seed [37,38], but these effects are primarily maternally determined via the development of the testa.

Previously, Moreau et al. [30] reported the value of an allelic series of mutants at the *r* locus for extending the range of natural variation and potentially improving the nutritional status of the pea. The data reported here relate to a further 17 isolines of the pea with mutations at five additional loci, providing a molecular description of a unique suite of pea lines for research and industrial uses. The mutants described here demonstrate the value of mutagenesis in generating variation for storage product content and composition in peas. More importantly, the effect on starch, most notably in the wrinkled-seeded mutants, has numerous pleiotropic effects, both on the development of the seed through an increase in the sugar content of cotyledons [32,39] and on other storage components, most notably lipid [40,41] and protein content and composition [14,42], that could be useful for industrial processing [10,42]. Other downstream effects of genetic variation in starch have also been observed, such as those on fibre content and vitamin C levels [43].

An original set of 32 induced mutant lines identified on the basis of seed shape, starch content or composition, and allelism tests [5] has been reduced to 25 unique alleles as a result of sequencing the genes encoding the respective proteins and identifying the specific mutations. Several of the original mutants duplicated other mutations at the same locus and some were likely to represent contaminants, possibly introduced during screening and isoline production (as suggested by SIM55 having an identical insertion to that in the naturally occurring *r* mutant [30] and additional ‘derived’ mutants having the naturally occurring *rb* mutation). Further explanation for the reduction in the number of original lines comes especially from the mutants at the *lam* locus. Two mutant lines—SIM502 and SIM512—listed originally as independent lines [5], were found to be identical alleles (*lam-b*) although they came from independent mutation experiments, one of which was to optimise the mutagen dose [34] before the main mutagenesis was undertaken. These were clearly EMS inductions of the same mutation. A further pair, SIM503 and SIM504, noted as potential siblings in the original screening, were confirmed as such through sequencing. These data are consistent with previous studies of *Lotus japonicus,* where some multiple mutations at the same site were observed (Supplementary Tables in Perry et al. [44]). However, the proportion of G to A transitions described here is very high compared with the general population (52%) used in that study, although it is much closer to that of the nodulation population bearing mutant phenotypes (73%; [44]).

Enzymes involved in starch synthesis either supply substrate to or create and elaborate the starch polymers, with *rug-4* (SUS1), *rug-3* (pPGM), and *rb* (AGPL1) falling into the former group and *lam* (GBSS1), *r* (SBEI), and *rug5* (SS2) falling into the latter [45]. The effect of mutations in genes encoding the substrate suppliers shows that, as the starch increases, the amylose content increases [45]. For the *rug3* mutants, there is also a good correlation between the effect of the mutations on protein structure and their starch phenotype. The *rug3-d* and *rug3-e* alleles are predicted to prevent activity since they have premature stop codons and, consequently, they have no starch (reported previously as 1% seed dry weight, the limit of the enzyme assay) [21,45] or visible starch granules. Similarly, *rug3-b* is predicted from modelling to adversely affect the catalytic activity of the enzyme, and, correspondingly, the mutant has no starch. The locations of the mutations in *rug3-a* (12% starch per unit seed dry weight) and *rug3-c* (5% starch per unit seed dry weight) alleles may impact substrate binding and catalysis but clearly do not prevent it as both mutants contain some starch. For the *rug4* locus, the *rug4-c* allele (which contains a premature stop codon) will interfere with substrate supply, providing a reference point for the effect of this locus, since it has been observed that no SUS1 protein is present [23]. However, mutants at this locus have the weakest effect on starch since other SUS isoforms probably have the capacity to supply sucrose; this isoform has its greatest effect where it is most highly expressed—in the root nodule—preventing nitrogen fixation in severe mutants [22]. The remaining two alleles are predicted through modelling to have similar effects since they are likely to destabilise the protein even though the protein is present [23]. The fact that all three alleles have similar effects on starch is consistent with conclusions based on the structural models.

The effects of alleles at the *rb* locus on the functionality of the AGPase enzyme is less clear. There has been much research on the functioning of this enzyme [46], and *Rb* encodes one subunit, AGPL1, of the heterotetrameric enzyme. The *rb-f* (early stop-codon) mutant would be the reference point, but the largest decrease in starch is normally observed in the naturally occurring *rb* mutant by a few percent. However, *rb-d* and *rb-h* mutants have similar phenotypes to *rb-f* indicating that all the mutations interfere with enzyme functionality, but none of the mutations prevents starch synthesis, unlike those at the *rug3* locus and other ADPGase mutants in potato and Arabidopsis [46].

For the polymer-forming enzymes reported here, the mutations show little variation for starch content in contrast to those reported previously at the *r* locus [30]. All *lam* alleles have a similar effect on starch and amylose and all are predicted through modelling to have a significant impact, reflecting their reduction in amylose to a very low level [25]. Similarly, all *rug5* mutations are predicted to disrupt the folding of the protein, and all have similar effects on starch amount and composition [45].

The screens that detected all the mutants outlined here involved the shape of the seed or the colour of starch grains. If starch is decreased sufficiently (or sucrose increased sufficiently) the seed becomes wrinkled [32]. The converse is not true—not all wrinkled seeds are affected in starch accumulation, as additional seed shape mutants were isolated in the screen that did not affect starch [5], and many are known that affect the seed coat alone, as mentioned earlier. The fact that multiple alleles for proteins involved in starch biosynthesis were isolated at each wrinkled-seeded locus, however, means that the screen was saturated. We did not detect mutants for other enzymes or subunits of AGPase that caused wrinkled seeds, meaning it is likely that such mutants alone would not have a significant impact on the amount of starch, just as the *lam* mutants could not be detected in the initial screen for seed shape and required a separate one for grain colour. Although a wrinkled-seeded phenotype was reported as a consequence of a reduction in the expression of the small subunits of AGPase in transgenic pea plants [47], we conclude that this reflects a simultaneous reduction in both subunits resulting from RNA interference constructs; the generation of TILLING mutants in both small subunit genes and the combination of mutant alleles would be needed to validate this conclusion.

Although the primary impact of the mutations described here is on seed shape and starch biosynthesis, the additional impact on protein [14] is relevant to industrial use, particularly where the fractionation of protein and starch fractions can be enhanced and the difference between the size and shape of starch granules and protein bodies can be exploited for ease of separation. Most notable are the most severe *rug3* mutations (*rug3-b*, *rug3-d*, *rug3-e*), where starch synthesis in seeds has been abolished completely, and can be used directly for protein. Additionally, the demand for contrasting food or feedstuffs can be met by choosing from the mutant pea lines described here. For example, this includes resistant versus highly digestible starches for food and feed, respectively, as well as low versus high amylose starches for differential processing.

The ease with which breeding programmes can incorporate such mutant lines is greatly enhanced by the availability of molecular tools for the identification of the precise mutation in segregant lines, such as, for example, using competitive allele-specific (KASP™) assays to enable bi-allelic scoring of single nucleotide polymorphisms; the sequence data for the mutants provided here enables such approaches. Equally, the combination of the starch-induced mutations with those impacting positively on protein quality—for example, mutations leading to the loss of trypsin inhibitors, lectin, albumin 2, and other so-called antinutritional proteins [13], and/or potential allergens such as vicilins [31]—has the potential to provide valuable additional sources of enhanced meat-free protein for novel foods. The incorporation of pea seeds and their products into a greater array of foods and at higher volumes than currently is supported further by research aimed at impacting taste and flavour [48]. Recently, progress has been made by targeting specific pathways using CRISPR/Cas9 gene editing technologies [49,50]. For example, this includes a reduction of major saponins that are negatively associated with consumer acceptability in pea seeds [51].

## 4. Materials and Methods

### 4.1. Plant Materials

The pea mutants were generated by an EMS/MNU mutagenesis programme [34] and were backcrossed six times to the parental line (BC1/RR) to create a set of near-isogenic lines. The original population was screened either for wrinkled-shaped seeds (*rugosus* lines; [34]) or for iodine-staining characteristics of starch granules (*lam* lines; [25]). The lines reported here are those that have been shown via sequencing to represent unique mutations at each locus, and are hence fewer than in the original published set based on phenotype and crossing [5].

### 4.2. Detection of Mutations

RNA was isolated from young frozen leaflets using lithium chloride and reverse-transcribed using Superscript II (Invitrogen, Thermo Fisher Scientific, FEI UK, Altringham, UK) to generate cDNA, as described previously [30,52] for the study of *r* and *rb* mutants. Genomic DNA was isolated from young leaflets of all mutant lines, as previously described [30,38]. A set of primers (Supplementary Table S1) was designed based on the NCBI/GenBank sequences for the respective wild-type pea gene or mRNA and, prior to a pea genome assembly being available (Supplementary Figure S1), the orthologue in *Medicago truncatula* (Mt) was used to predict intron/exon structure (*P. sativum* mRNA for starch-branching enzyme I: X80009.1 (*r*); *P. sativum* mRNA for ADP-glucose pyrophosphorylase large subunit1: X96766.1 (*rb*); *P. sativum* partial mRNA for plastidial phosphoglucomutase: AJ250770, and Mt AC148527 gene (*rug3*); *P. sativum* mRNA for sucrose synthase: AJ012080, and GCA_900700895.2 gene Psat4g019440 (*rug4*); *P. sativum* mRNA for starch synthase: X88790.1 (*rug5*); *P. sativum* mRNA for granule-bound starch synthase 1: XM_051037336.1 and GCA_900700895.2 gene Psat0s4284g0040 (*lam*)). Following PCR, amplicons were sequenced, using forward and reverse primers (Eurofins Genomics, Ebersberg bei München, Germany).

Where fluorescently labelled primers were used in analyses of *rb* size variants, the gene-specific forward primer for PCR was designed to include a M13 tail and a fluorescent label at its 5′ end (M13_TGTAAAACGACGGCCAGT_FAM or Hex fluorescent label). Here we show data based on using PsAGPL1_F1 and PsAGPL1_R8, where the former includes the fluorescently labelled tail. Amplicons were separated and detected, using an Applied Biosystems DNA analyser 3730XL (Thermo Fisher Scientific, FEI UK, Altringham, UK), and their sizes were determined relative to fluorescent markers, using GeneMarker v2.6 software (SoftGenetics State College, PA 16803 USA).

### 4.3. Protein Structure Modelling

To provide a three-dimensional framework for the interpretation of our phenotypic data, we predicted the structures of the corresponding gene products. This allowed us to speculate on the likely impact of the mutations on enzymatic function. Prior to modelling, the predicted target peptide sequences were removed from the input sequences based on outputs from the TargetP 2.0 prediction tool accessed on 26 March 2024 [53,54]). Models were computed via the ColabFold v1.5.2 notebook [55,56], utilising the protein structure prediction tool AlphaFold2 accessed on 26 March 2024 [57]. The quality of the resultant models was assessed via the pLDDT scores and PAE plots provided alongside these models. With the exception of some N- and/or C-terminal tails and surface loops, the models were of high quality. To establish any potential biological effects of the identified mutation sites, the AlphaFold2 models were compared to related experimentally determined structures retrieved using the PDBeFold [58] and AlphaFill [59] servers, both accessed on 10 April 2024, with the latter specifically finding structures with bound ligands. In some cases, these comparisons helped in identifying whether the mutations occurred in catalytic residues or those important for substrate binding. The structures were superposed and images were generated using ChimeraX 1.7.1 [60].

## 5. Conclusions

Pea represents an important legume for human consumption and animal feed, but its genetic diversity has not been well exploited. The 27 mutant lines, for which a full molecular characterisation is reported here, show that a wide range of starch (and protein) materials can be achieved in pea, from low to high amylose, as well as simple, complex, or super-complex granules, and low to high protein content, including starch-free protein. The data presented here will permit a range of mutations to be incorporated readily into elite varieties to accelerate the development of pea as a major starch and protein crop of the future. Furthermore, the novel and precise information presented on the impact of the mutations can be used to guide and inform future mutagenesis approaches, particularly gene editing approaches where starch variation is being sought in pea and other crops.

## Figures and Tables

**Table 1:** Mutations in unique alleles of starch mutants. The positions of the mutations are cited relative to the initiator methionine of genomic (ATG codon, Supplementary Figure S1) and encoded protein sequences, using gene sequences retrieved from the JI2822 *(rbrb*) genome assembly for pea [31].

Allele	Isolation Number (Prefix − E = EMS; M = MNU)	Line Number †	Mutation		
			Position	Genomic	Protein
*r*	N/A	BC1/rr	Exon 22	A27379-insertion of ~1 kb ‘element’	Predicted E 860 →, ≤30 aberrant amino acids *
*r-c*	M(850)7065	SIM53	Exon 15	G22948A	R 571 H
*r-d*	M(718)7339	SIM54	Exon 13	G20772A	G 495 D
*r-f*	E499(1677)	SIM56	Exon 18	G25551A	W 684 *
*r-g*	E(117)22	SIM57	Exon 18	G25633A	R 712 C
*r-h*	E(349)7277	SIM58	Intron 15 acceptor splice site	G24153A	10 aberrant amino acids: 586–595 ^1^*
*r-i*	E(625)113	SIM59	Intron 8 acceptor splice site	G13242A (84 nucleotide deletion: 957–1040)	28 amino acid deletion (319–346) ^2^
*r-j*	E(122)1154	SIM61	Exon 16	G24218A	E 607 K
*r-k*	M(32)7325	SIM71	Intron 16 donor splice site	G24299A	Variants include: loss of 13 amino acids (621–633) ^3^; four aberrant amino acids (634–637) *
*rb*	N/A	BC2/rbrb	Exon 2	T470-G471 (9 bp deletion)	A98-(ATP or TPA missing)-A99
*rb-d*	E(162)1232	SIM15	Exon 13	G2802A	C 456 Y
*rb-f*	E(1663)5142	SIM101	Exon 8	G1924A	W 304 *
*rb-h*	E(1258)3892	SIM103	Exon 4	G1153A	G 174 R
*rug3-a*	E(543)1836	SIM1	Exon 4	G703A	G 128 D
*rug3-b*	E(626)124	SIM32	Exon 20	C4367T	R 567 C
*rug3-c*	E(476)1589	SIM41	Exon 7	G1479A	G 195 S
*rug3-d*	E(549)548	SIM42	Exon 4	C711T	R 131 *
*rug3-e*	E(1780)5702	SIM43	Exon 17	CT deletion 3757/3758	S 477 C, 479 *
*rug4-a*	E(620)121	SIM11	Exon 4	C788T	L 164 F
*rug4-b*	E(77)16	SIM91	Exon 11	G2668A	R 578 K
*rug4-c*	M(3)545	SIM201A	Intron 8 donor splice site	G1966A	C 432 I, 37 aberrant amino acids, 468 *
*rug5-a*	E(640)2239	SIM51	Exon 8	G3702A	W 708 *
*rug5-b*	E(167)1236	SIM52	Exon 3	G1926A	G 281 R
*rug5-c*	E(509)1728	SIM81	Exon 8	G3653A	G 692 D
*lam-a*	E(1470)4322	SIM501	Exon 1	G253A	E 85 K
*lam-b*	E(0.3)151 ^4^/ E(722)149	SIM502/512	Intron 6 acceptor splice site	G1654A	A 254 R, 12 aberrant amino acids, 266 *
*lam-c*	E(1599)4812	SIM503/504	Intron 1 acceptor splice site	G454A	G 108 V, 11 aberrant amino acids, 119 *

* indicates premature stop codon. † SIM numbering from Wang & Hedley [33]. ^1^ FCVNGSNCHP. ^2^ EKYVFKHPQPKRPQSIRIYESHIGMSSP. ^3^ VGDKTLAFWLMDK. ^4^ from a separate mutagenesis experiment.

## Data Availability

All the pea lines described here have been deposited to, and will be publicly available from, the *Pisum* Collection at the Genetic Resources Unit, John Innes Centre (https://www.seedstor.ac.uk), upon publication of this work.

## Supplementary Materials

The following supporting information can be downloaded at: https://www.mdpi.com/article/10.3390/ijms26062612/s1.
