# Supplementary material for: A Suite of Pea (Pisum sativum L.) Near-Isolines: Genetic Resources and Molecular Tools to Breed for Seed Carbohydrate and Protein Quality in Legumes
Source: Int J Mol Sci. 2025 Mar 14;26(6):2612. doi: 10.3390/ijms26062612 (PMC11942445; doi:10.3390/ijms26062612)
Supplement: Supplementary file 1 [file ijms-26-02612-s001.zip › ijms-3410026-Supplementary Figure S1.pdf]

Supplementary Figure S1

A. Mutations at *rb*: ADP-glucose pyrophosphorylase 1 large subunit (AGPL1; Agpl1 in alignment) chr5:637125150-637122112

```
                  Exon 1
Agpl1             ATGGCTTCTGGTTGTGTGAGCTTGAAAACCAACACCCATTTTCCAAATTCTAAAAAAGGT 60
5g109520          ATGGCTTCTGGTTGTGTGAGCTTGAAAACCAACACCCATTTTCCAAATTCTAAAAAAGGT 60
                  ************************************************************

Agpl1             TCTTTTTTTGGGGAAAGAATCAAAGGAAGCTTGAAAAACAGTTCATGGGTCACTACCCAG 120
5g109520          TCTTTTTTTGGGGAAAGAATCAAAGGAAGCTTGAAAAACAGTTCATGGGTCACTACCCAG 120
                  ************************************************************

Agpl1             AAGAAGATCAAACCTGCTTCTTTTTCTGCTATTCTTACTTCAGATGACCCCAAAGGTTCC 180
5g109520          AAGAAGATCAAACCTGCTTCTTTTTCTGCTATTCTTACTTCAGATGACCCCAAAGGTTCC 180
                  ************************************************************
                  Intron 1
Agpl1             CTGGTAAACTCAGTTTCATTCTGGGTTTCACTTTTTGCTTCCAATTCTGAAAAAAAGAAA 240
5g109520          CTG--------------------------------------------------------- 184
                  ***

Agpl1             GACTTTTTTTTTCTCCCATTATATGACATAACTTTTTTATGTTAATTATTTTGCTACATT 300
5g109520          ------------------------------------------------------------ 184


Agpl1             TTGTTTGGTATATGATTATGATTATGATTATGATTTTGAGTGTATGTTTTGAAATTCAGA 360
5g109520          -----------------------------------------------------------A 184
                                                                             *
                  Exon 2
Agpl1             ATTTGCAAGTGCCTTCATTTCTGAGACTAAGAGCTGATCCAAAAAATGTGATTTCCATTG 420
5g109520          ATTTGCAAGTGCCTTCATTTCTGAGACTAAGAGCTGATCCAAAAAATGTGATTTCCATTG 244
                  ************************************************************
                                              Deleted 9 bp in rb_GCAACACCT Intron 2
Agpl1             TGTTGGGAGGAGGGCCTGGAACACATCTCTATCCTCTTACCAAACGAGCT GCGGTGAGTG 480
5g109520          TGTTGGGAGGAGGGCCTGGAACACATCTCTATCCTCTTACCAAACGAGCT GCG------- 298
                  ************************************************** ***

Agpl1             AGTTTTATGATATGAATTGTTGTCTGAATTCTAACATTTTGGTGTTGTTTGTAACTTTGT 540
5g109520          ------------------------------------------------------------ 298

                                                              Exon 3
Agpl1             GCTGTTTGTGATGAAGATTTGTTTGCTGTGTTTGTTTTGAAGGTTCCTGTTGGAGGATGC 600
5g109520          ------------------------------------------GTTCCTGTTGGAGGATGC 315
                                                            ******************

Agpl1             TATAGGCTTATAGACATTCCAATGAGCAACTGCATCAATAGTGGCATCAACAAGATATTT 660
5g109520          TATAGGCTTATAGACATTCCAATGAGCAACTGCATCAATAGTGGCATCAACAAGATATTT 375
                  ************************************************************

Agpl1             GTGCTGACTCAGTTCAACTCTGCTTCACTAAATCGTCACATCGCTCGCACCTATTTCGGA 720
5g109520          GTGCTGACTCAGTTCAACTCTGCTTCACTAAATCGTCACATCGCTCGCACCTATTTCGGA 435
                  ************************************************************
                                                          Intron 3
Agpl1             AATGGTGTCAACTTTGGAGATGGATTTGTGGAGGTAATCATGCATCACCATCATGAAAAT 780
5g109520          AATGGTGTCAACTTTGGAGATGGATTTGTGGAG--------------------------  465
                  *********************************

Agpl1             GGATGGAAATTAGTACTTTTTTTTTCTCGCATTTTTTATATTGGAACAATCTTTTAGCAT 840
5g109520          ------------------------------------------------------------ 465


Agpl1             ACTTTGTTTATCTTTTGGTCGAACTCCGAATAACTAGGATCCGTCTTTTCTGGGATTTAG 900
5g109520          ------------------------------------------------------------ 465


Agpl1             AGAGTTAAGAACAAAAAAATATATTTTCATGCAATGAGTTTTGAGATGAACATGACAGTT 960
5g109520          ------------------------------------------------------------ 465
```

```
Agpl1          GCCAACAAACAAAACATAAGTAATTCGGTTTGCGTAGAGAAATACCGATCCAGTGAAGGA 1020
5g109520       ------------------------------------------------------------ 465


Agpl1          GATTCTTGTTGATGAATTTCAAGTTTGTTTCTATTTATGATTCATGAGGACTAATTAAAG 1080
5g109520       ------------------------------------------------------------ 465

                                      Exon 4
Agpl1          TCAATCTAAATGTCAAAATAGGTTCTGGCGGCGACACAAACACCAGGAGAAGCTGGGAAG 1140
5g109520       ---------------------GTTCTGGCGGCGACACAAACACCAGGAGAAGCTGGGAAG 507
                                    ***************************************

                          rb-h                                    Intron 4
Agpl1          AAGTGGTTTCAAGGAACTGCAGATGCTGTGAGACAATTTACCTGGATATTTGAGGTAGAC 1200
5g109520       AAGTGGTTTCAAGGAACTGCAGATGCTGTGAGACAATTTACCTGGATATTTGAG------ 561
               ******************************************************

Agpl1          AAAGGATTTTTGTTGTTGTTGTATATACATTTCGATAAATAATAGATTCTTTTGTTCTCA 1260
5g109520       ------------------------------------------------------------ 561

                                                              Exon 5
Agpl1          TTTTTGAGCTTGTCAATAAGTAATAGATTGTTTGTGGTAGGATGCCAAGAATATAAACGT 1320
5g109520       ----------------------------------------GATGCCAAGAATATAAACGT 581
                                                       ********************

Agpl1          CGAGAATGTATTGATCTTGGCCGGAGATCATTTATATCGAATGGATTACATGGATCTATT 1380
5g109520       CGAGAATGTATTGATCTTGGCCGGAGATCATTTATATCGAATGGATTACATGGATCTATT 641
               ************************************************************
                Intron 5
Agpl1          GCAGGTATATTGTGAATGTTTTGTAGAGTGGATTGTTTTTCATTTCATGTTCTAGAGTTT 1440
5g109520       GCAG-------------------------------------------------------- 642
               ****
                                                         Exon 6
Agpl1          TCTGATTCATCTATATAACAAATTAACAGAGTCACGTTGATAGAAATGCCGATATTACAG 1500
5g109520       -----------------------------AGTCACGTTGATAGAAATGCCGATATTACAG 676
                                            *******************************
                                       Intron 6
Agpl1          TTTCGTGTGCTGCCGTTGGTGACAAGTGAGTATTATACTTTATTTCATTCTAGAATGGGT 1560
5g109520       TTTCGTGTGCTGCCGTTGGTGACAA----------------------------------- 699
               *************************

Agpl1          GAATAGAAAGAATCAACTAAGTGGAAACAAAATTACGATTTCCATTGTCTGATTTTCAGC 1620
5g109520       -----------------------------------------------------------C 702
                                                                           *
                Exon 7
Agpl1          CGCGCATCTGATTATGGATTGGTCAAAGTAGACGACAGAGGCAACATCGTACAATTTTCA 1680
5g109520       CGCGCATCTGATTATGGATTGGTCAAAGTAGACGACAGAGGCAACATCGTACAATTTTCA 762
               ************************************************************
                                                         Intron 7
Agpl1          GAAAAACCGAAAGGCGCTGATCTGAAAGCAATGGTAATTCCAAACATAATAACCTAATGT 1740
5g109520       GAAAAACCGAAAGGCGCTGATCTGAAAGCAATG--------------------------- 794
               *********************************

Agpl1          TTGCACCTTCTTTCTGTTTTCTTGTTATCTGGTAAATCCACTGAGATTGTTCATCCTCAT 1800
5g109520       ------------------------------------------------------------ 794

                       Exon 8
Agpl1          TTTGCAGCAAGTAGATACTTCTCGTCTTGGGTTGTCACCGCAAGACGCATTGAAGTCGCC 1860
5g109520       -------CAAGTAGATACTTCTCGTCTTGGGTTGTCACCGCAAGACGCATTGAAGTCGCC 848
                      *****************************************************

Agpl1          ATATATTGCATCTATGGGAGTTTATGTGTTCAAGAAAGATGTTTTACTCAAGCTTCTGAA 1920
5g109520       ATATATTGCATCTATGGGAGTTTATGTGTTCAAGAAAGATGTTTTACTCAAGCTTCTGAA 908
               ************************************************************
                rb-f
Agpl1          ATGGAGGTATCCTACTTCTAATGACTTCGGATCCGAAATCATTCCTTCCGCAATAAGAGA 1980
5g109520       ATGGAGGTATCCTACTTCTAATGACTTCGGATCCGAAATCATTCCTTCCGCAATAAGAGA 968
               ************************************************************
                              Intron 8
Agpl1          GCACAATGTCCAAGTAAGAGGAATTCCGATAAATATATCAGCTTACAAATGTTTTTATCC 2040
5g109520       GCACAATGTCCAA----------------------------------------------- 982
```

```
                **************
                                                              Exon 9
Agpl1           ATTTCAGAAGATTTTTATCTGCCATCTATGCTTTTATGCAGGCATATTTTTTCGGAGACT 2100
5g109520        -----------------------------------------GCATATTTTTTCGGAGACT 1000
                                                         *******************

Agpl1           ACTGGGAAGATATTGGAACGATAAAATCCTTCTACGATGCTAACCTCGCTCTTACTGAAG 2160
5g109520        ACTGGGAAGATATTGGAACGATAAAATCCTTCTACGATGCTAACCTCGCTCTTACTGAAG 1060
                ************************************************************
                 Intron 9
Agpl1           AGGTAGGTTCAAGAATTTTTCAGTGTTCTTGTTCAGTTTAGTTGATTGAAACTAAAATCT 2220
5g109520        AG---------------------------------------------------------- 1061
                **
                                       Exon 10
Agpl1           GCTACATGTTACTCTCTCACAGAGTCCAAAGTTCGAGTTTTATGATCCAAAAACTCCGAT 2280
5g109520        ----------------------AGTCCAAAGTTCGAGTTTTATGATCCAAAAACTCCGAT 1100
                                      **************************************
                                                             Intron 10
Agpl1           TTTCACATCTCCAGGATTCCTACCACCAACAAAGATTGACAACTCTCGGGTACGATAATC 2340
5g109520        TTTCACATCTCCAGGATTCCTACCACCAACAAAGATTGACAACTCTCGG----------- 1150
                *************************************************

Agpl1           TATCTATCTTGTATCATGCTAATAATTCGAAACATCATGTCTTTCTTATTCTGTCCGCGT 2400
5g109520        ------------------------------------------------------------ 1150

                                       Exon 11
Agpl1           TGCTTTGGTTTTGGTAGGTTGTGGATGCCATTATCTCCCATGGATGTTTCCTGAGAGATT 2460
5g109520        -----------------GTTGTGGATGCCATTATCTCCCATGGATGTTTCCTGAGAGATT 1192
                                 *******************************************

Agpl1           GTACAATCCAACACTCCATTGTAGGTGAAAGGTCGCGTTTAGATTATGGCGTTGAGCTTC 2520
5g109520        GTACAATCCAACACTCCATTGTAGGTGAAAGGTCGCGTTTAGATTATGGCGTTGAGCTTC 1252
                ************************************************************
                 Intron 11
Agpl1           AGGTAATTAAACTACCATATATTCTAGACTCGTTACTCCATTAAACGGTTCCTCCTAACG 2580
5g109520        AG---------------------------------------------------------- 1253
                **
                                       Exon 12
Agpl1           AAAAACAAGATCAACTTTTACAGGACACTGTAATGATGGGAGCTGACTATTACCAAACTG 2640
5g109520        -----------------------GACACTGTAATGATGGGAGCTGACTATTACCAAACTG 1291
                                       *************************************

Agpl1           AATCCGAAATCGCTTCCCTACTTGCAGAAGGGAAGGTCCCGATTGGCATCGGAAGGAATA 2700
5g109520        AATCCGAAATCGCTTCCCTACTTGCAGAAGGGAAGGTCCCGATTGGCATCGGAAGGAATA 1351
                ************************************************************
                        Intron 12
Agpl1           CCAAAATCAAGTAAAACCTTAGCATTTTCGCGTAGAAAATCTTCCTTTTTACTATTGTGA 2760
5g109520        CCAAAATCAA-------------------------------------------------- 1361
                **********
                                              rb-d Exon 13
Agpl1           AAACTCATAGTTTGTTATTTATTATCTCTGTTAAAGGAACTGCATTATTGACAAGAATGC 2820
5g109520        ------------------------------------GAACTGCATTATTGACAAGAATGC 1385
                                                    ************************
                                              Intron 13
Agpl1           AAAAATCGGGAAAGAAGTTGTCATCGCGAACAAAGAAGTAAGAATAAAACAAGTTAGTAG 2880
5g109520        AAAAATCGGGAAAGAAGTTGTCATCGCGAACAAAGAA----------------------- 1421
                *************************************
                                                             Exon 14
Agpl1           TTTTCCGTTCCACGATACATTTGTTCACGCGTATAAACTCGAATGCTTTATCTGTAGGGC 2940
5g109520        ---------------------------------------------------------GGC 1425
                                                                         ***

Agpl1           GTTCAAGAAGCAGATAGATCGGAAGATGGTTTCTACATCCGATCAGGAATCACCATCATA 3000
5g109520        GTTCAAGAAGCAGATAGATCGGAAGATGGTTTCTACATCCGATCAGGAATCACCATCATA 1485
                ************************************************************

Agpl1           ATGGAGAAAGCAACGATAGAAGACGGAACTGTCATATAA       3039
5g109520        ATGGAGAAAGCAACGATAGAAGACGGAACTGTCATATAA       1524
                ***************************************
```

Mutant	F primer for screening	R primer for screening
rb	(F1) GCAAGTGCCTTCATTTCTGAG	(R5) GCATCCTCCAACAGGAACC
rb-d	GAATCCGAAATCGCTTCCC	CGATGACAACTTCTTTCCCG
rb-f	GCAAGTAGATACTTCTCGTCTTGG	ACATTGTGCTCTCTTATTGCGG
rb-h	TGGAGATGGATTTGTGGAGG	TGAACACATAAACTCCCATAGATGC

B. Mutations at *rug3*: phospoglucomutase (pPGM)
chr6:449387170-449391892

```
                   Exon 1
pPGM               ATGGCTTTCTGTTACAGACTCGACAACTTCATCATCTCTGCGTTTAAACCCAAACACTCA     60
6G70620            ATGGCTTTCTGTTACAGACTCGACAACTTCATCATCTCTGCGTTTAAACCCAAACACTCA     60
                   ************************************************************

pPGM               AATGTCCCACTTTCAATTCATCATTCATCATCCAATTTTCCTTCTTTCAAAGTTCAAAAC     120
6G70620            AATGTCCCACTTTCAATTCATCATTCATCATCCAATTTTCCTTCTTTCAAAGTTCAAAAC     120
                   ************************************************************

pPGM               TTTCCTTTCAGGGTTCGCTATAATTCAGCTATTAGAGCCACTTCGTCTTCCTCTTCTACT     180
6G70620            TTTCCTTTCAGGGTTCGCTATAATTCAGCTATTAGAGCCACTTCGTCTTCCTCTTCTACT     180
                   ************************************************************
                                                     Intron 1
pPGM               CCCACAACCATTGCAGAACCTAATGACATTAAGGTTTTTTTTCCCTTCACACATTTATTT     240
6G70620            CCCACAACCATTGCAGAACCTAATGACATTAAG---------------------------     212
                   *********************************

pPGM               CTATTTTTATGTTTTTAAAAAAAATTAATTTAACCCTTTTGAATGTTTTTTATTTTTGTG     300
6G70620            ------------------------------------------------------------     212

                          Exon 2
pPGM               TGAAGATTAACTCTATTCCTACTAAACCTATTGAAGGACAAAAAACTGGTACCAGTGGCC     360
6G70620            -----ATTAACTCTATTCCTACTAAACCTATTGAAGGACAAAAAACTGGTACCAGTGGCC     268
                        *******************************************************
                                  Intron 2
pPGM               TAAGAAAAAAGGTTGGTATTTGTTCTATTCATATTCAACCCTTTGATTCATTATGTTTGA     420
6G70620            TAAGAAAAAAG-------------------------------------------------     280
                   ***********

pPGM               TCAAAATGCTCACATTATGATTGATGTTAATATATTTGTGCATGCAAAATGAAATTGTGT     480
6G70620            ------------------------------------------------------------     280

                                              Exon 3
pPGM               TTGTTTATATATGCTGAATTGTTTTGTAGGTGAAAGTGTTTAAGCAAGAAAATTACCTTG     540
6G70620            -----------------------------GTGAAAGTGTTTAAGCAAGAAAATTACCTTG     310
                                                ******************************
                                  Intron 3
pPGM               CAAATTGGATTCAGGTTTGTAGAATCCCATGCAATGTGTAAAGATTATATGGTTAATGTG     600
6G70620            CAAATTGGATTCAG----------------------------------------------     322
                   **************
                                                                 Exon 4
pPGM               TTTTTGCTTTGTAATGTAAGTTAAACACTTATGTGGATTTGTAGGCACTGTTTAATTCGT     660
6G70620            --------------------------------------------GCACTGTTTAATTCGT     340
                                                               ****************
                                                            rug3-a   rug3-d
pPGM               TGCCGCCGGAGGATTACAAGAATGGATTGTTGGTTTTGGGAGGCGATGGTCGATACTTCA     720
6G70620            TGCCGCCGGAGGATTACAAGAATGGATTGTTGGTTTTGGGAGGCGATGGTCGATACTTCA     400
                   ************************************************************
                                         Intron 4
pPGM               ATAAAGAAGCTGCACAGGTCAAATTTTTTGAATGTGTCAACTGAATCTTGTGTTAAGAAA     780
```

```
6G70620         ATAAAGAAGCTGCACAG-------------------------------------------          420
                *****************
                                                                    Exon 5
pPGM            ACGGCTTGATTTCTAATTTGTCGGCTTGTTTTTTTGTGTGATTTGCAGATAATAATCAAG          840
6G70620         ------------------------------------------------ATAATAATCAAG          429
                                                                ************
                                                             Intron 5
pPGM            ATTGCTGCTGGAAATGGTGTTGGAAAAATTCTGGTTGGGAAGTAAGTTTCGATTGATTTT          900
6G70620         ATTGCTGCTGGAAATGGTGTTGGAAAAATTCTGGTTGGGAA-------------------          468
                *****************************************

pPGM            GGTTTATTGAATTAGTTGCAACAAACAAGATGAATCACTTAAATCAAGAGCTAGTAGGGA          960
6G70620         ------------------------------------------------------------          468


pPGM            TTGTCATTGATTCGATAAAACAAAACATCTTTCTTGGATTAGTGCATGAGCTATTGTTCA          1020
6G70620         ------------------------------------------------------------          468


pPGM            ATGCAATCTGTTTGGAATGACTGATTTAAGTTTATCTGCTGACATAAGTACTTGTAAGAC          1080
6G70620         ------------------------------------------------------------          468


pPGM            TTTTTGTTTGGGAGGACTTGTAGAAACAACTTACGGCGTATTCATAAGTTGTTTTGAGCT          1140
6G70620         ------------------------------------------------------------          468


pPGM            TATTTTCATGATAGCTTAAAAAAATAACTTATAGCTTATACGGAAACAATTTGACTTTAT          1200
6G70620         ------------------------------------------------------------          468


pPGM            TTGATCTTTTGTTATAAAAGTAACTAAGTTGTTTATCCAAACAAAGCGTAAATTGAATAC          1260
6G70620         ------------------------------------------------------------          468

                                      Exon 6
pPGM            GCGTTTTCTATTCTAAATTCAGGGAAGGGATATTGTCAACGCCAGCCGTTTCTGCTGTGA          1320
6G70620         ----------------------GGAAGGGATATTGTCAACGCCAGCCGTTTCTGCTGTGA          508
                                      **************************************
                               Intron 6
pPGM            TAAGGAAGAGAGAGGCAAGCAAAACTAACTCCTTTTTCTCAGGCATAACATTTTCATTCA          1380
6G70620         TAAGGAAGAGAGAG----------------------------------------------          524
                **************
                                                     Exon 7
pPGM            ACTTTCTCTTTTCTTCTCATTTTCTTAAACTTCAACAGGCAAATGGTGGGTTTATCATGA          1440
6G70620         --------------------------------------GCAAATGGTGGGTTTATCATGA          544
                                                      **********************
                                                     rug3-c   Intron 7
pPGM            GTGCGAGCCATAACCCTGGTGGACCTGAATATGATTGGGGTATTAAGGTGACTTTCCTAA          1500
6G70620         GTGCGAGCCATAACCCTGGTGGACCTGAATATGATTGGGGTATTAAG-------------          589
                ***********************************************

pPGM            TTAATTCTCATCAATTAGAAGAGGATTCTGTCATTTTCTATCTAATGTACATGTTAAGAT          1560
6G70620         ------------------------------------------------------------          589


pPGM            ATGTGGTTGTGCCTAACTCAACTATAACATGGTATCAGAGTCTGGTTCGGCGTAATGATG          1620
6G70620         ------------------------------------------------------------          589


pPGM            AGTTAGGCCTAACCACATTTCCTTGGATCGGAATCAGGCTCTGATACCATATCAAGAATG          1680
6G70620         ------------------------------------------------------------          589


pPGM            TGTGGTTGGGTCTAACTCTAACTCTAACTCTAATAGTACATAATGAATACCGATTAGTCA          1740
6G70620         ------------------------------------------------------------          589


pPGM            ATTCATGGTTTTTTCTGTAACGTGTCTGTACTTTGTAGTTAATGTTGAGAGACTGTGTTA          1800
6G70620         ------------------------------------------------------------          589

                                                                    Exon 8
```

```
pPGM           TGTGCAAATCTATCGGTTGGTGTTACTGATAAATTTCTTTTCTGCTGAATTCAGTTTAAT     1860
6G70620        ------------------------------------------------------TTTAAT     597
                                                                     ******

pPGM           TACAGTAGCGGACAACCTGCACCAGAATCCATCACCGACAAGATTTACGGAAACACCCTA     1920
6G70620        TACAGTAGCGGACAACCTGCACCAGAATCCATCACCGACAAGATTTACGGAAACACCCTA     657
               ************************************************************
                      Intron 8
pPGM           TCTGTAAGTGTCGCATTCAAGTATGCCTTAATATTCCGATCCTACCTTCTGCACAGTGGA     1980
6G70620        TCT---------------------------------------------------------     662
               ***
                                                            Exon 9
pPGM           TACTTTTCTCAGCTTTTTCATACAATTTTCATTGACACAGATTTCTGAGATAAAGATTGC     2040
6G70620        ----------------------------------------ATTTCTGAGATAAAGATTGC     680
                                                       ********************

pPGM           TGATATTCCCGACGTTGACTTATCAAATGTTGGAGTTACGAAATTCGGAAGCTTCAGTGT     2100
6G70620        TGATATTCCCGACGTTGACTTATCAAATGTTGGAGTTACGAAATTCGGAAGCTTCAGTGT     740
               ************************************************************
                                                            Intron 9
pPGM           GGAAGTAATTGACCCAGTTTCTGATTACCTGGAGTTATTAGAGGTCAAAATAATTTGTTT     2160
6G7062         GGAAGTAATTGACCCAGTTTCTGATTACCTGGAGTTATTAGAG-----------------     781
               *******************************************

pPGM           TCATTATTATAATCCAGTAGAATGATTCATGCAAGCTCGGCCTCAGTGTTGTGAGTTTTA     2220
6G70620        ------------------------------------------------------------     781

                                                            Exon 10
pPGM           TTTCATTTCATTTCAGTTATTTGCTATTATTTTTTGTGCAGACAGTGTTCGATTTTCAGC     2280
6G70620        -----------------------------------------ACAGTGTTCGATTTTCAGC     802
                                                        *******************
                                                     Intron 10
pPGM           TAATCAAAAGTCTTATTTCACGGCCAGATTTTAGGTACAACCTTAACATTTATTCCACCA     2340
6G70620        TAATCAAAAGTCTTATTTCACGGCCAGATTTTAG--------------------------     835
               **********************************

pPGM           TAATCCCAATCTTGGCACATTGTCTCGAAACTGATTGAGTTCTTTAAATTTTTTATTTTA     2400
6G70620        ------------------------------------------------------------     835

                      Exon 11
pPGM           GGTTTACATTTGATGCCATGCATGCCGTTGCTGGTGCTTATGCAACACCCATTTTCGTTG     2460
6G70620        -GTTTACATTTGATGCCATGCATGCCGTTGCTGGTGCTTATGCAACACCCATTTTCGTTG     895
                ***********************************************************
                                     Intron 11
pPGM           ATAAACTTAGTGCTAGTCTGGTATGGTTCTTCCCCTTTTACTCTTGTACGGAGTAGGTAC     2520
6G70620        ATAAACTTAGTGCTAGTCTG----------------------------------------     917
               ********************
                                                                   Exon 12
pPGM           AAATCTATACGGTGTTGTTGTTGATTTTCGGTGTTATGTATGCAATGTAGGATTCAATTT     2580
6G70620        --------------------------------------------------GATTCAATTT     925
                                                                 **********
                                                                  Intron 12
pPGM           CAAATGGAATACCTTTGGAAGATTTTGGACACGGTCATCCTGATCCTAATCTAACGTGAG     2640
6G70620        CAAATGGAATACCTTTGGAAGATTTTGGACACGGTCATCCTGATCCTAATCTAAC-----     981
               *******************************************************

pPGM           TTTAGTTTTATCTTTTCAACATTGTGTTTTCAATCATTAGTAAATTGTTTTTGATTCTAA     2700
6G70620        ------------------------------------------------------------     981

                              Exon 13
pPGM           TGTTTATTGAACAGATACGCAAAGGATCTTGTCAAGATTATGTATGCTGAAAACGGACCT     2760
6G70620        --------------ATACGCAAAGGATCTTGTCAAGATTATGTATGCTGAAAACGGACCT     1026
                             **********************************************
                                     Intron 13
pPGM           GATTTTGGTGCCGCTAGTGATGGTATGCGAGATTTAAGTTATTTTTGAAATTTAACTTGT     2820
6G70620        GATTTTGGTGCCGCTAGTGATG----------------------------------         1052
               **********************

pPGM           TTCTGTTGATAAATCCTTTTGCAACAATGTTTTGTCTGAACTCAAGAGCAATTGGATGAG     2880
6G70620        ------------------------------------------------------------     1052
```

```
pPGM            ATGGTAAGAGATCTCTTTCAGTTTAACCTGAGGTCCTGGGTTTGAACTCAGCCCCGGGCA       2940
6G70620         ------------------------------------------------------------       1052


pPGM            TGCGACAGTGCTAAATTCTCTTGAGGGAGAGCTTTGCCGCTCATTGCGGTCCTCCCAGCT       3000
6G70620         ------------------------------------------------------------       1052


pPGM            CGAGGAATTAGTCTCTGCAGTTGCGCGCAGAGGATACCCGATTTTTACTGTAAAAAAACA       3060
6G70620         ------------------------------------------------------------       1052


pPGM            ATGTTTTTTGTCTGCATTTGTTTACTTGATAATGTTTATGTATTTTAACTTTCGTTTAGG       3120
6G70620         -----------------------------------------------------------G       1052
                                                                           *
                Exon 14
pPGM            TGATGGTGATAGAAATATGATTTTGGGAACAAGTTTCTTCGTAACTCCTTCAGACTCTGT       3180
6G70620         TGATGGTGATAGAAATATGATTTTGGGAACAAGTTTCTTCGTAACTCCTTCAGACTCTGT       1109
                ************************************************************

pPGM            AGCCGTTATTGCAGCCAATGCAAAAGAAGCGATTCCGTACTTTAAGGACAGTATCAAGGT       3240
6G70620         AGCCGTTATTGCAGCCAATGCAAAAGAAGCGATTCCGTACTTTAAGGACAGTATCAAG--       1166
                **********************************************************
                Intron 14
pPGM            AGAAAGTTTGTGCATATCATATTATTCACAAGTATTCGTTGTTGTAAAACAGAAGTGTCA       3300
6G70620         ------------------------------------------------------------       1166

                                      Exon 15
pPGM            TTGTTCTGTATTGTAATTGCAGGGTCTTGCACGATCAATGCCGACAAGCGGTGCTCTAGA       3360
6G70620         ----------------------GGTCTTGCACGATCAATGCCGACAAGCGGTGCTCTAGA       1205
                                      **************************************
                                                     Intron 15
pPGM            TAGAGTTGCTGAAAAGTTGAACCTCCCTTTTTTTGAGGTATAGTATGATTTTACATTGTT       3420
6G70620         TAGAGTTGCTGAAAAGTTGAACCTCCCTTTTTTTGAG-----------------------       1241
                *************************************

pPGM            GTTGCGTTTAGAATTATTCTAGATTATGATTCTCGAATGACAATAGACGGACCTCGGGTG       3480
6G70620         ------------------------------------------------------------       1241

                       Exon 16
pPGM            CAGGTTCCCACTGGTTGGAAATTCTTTGGTAATCTTATGGATGCTGGAAATCTGTCGATT       3540
6G70620         ---GTTCCCACTGGTTGGAAATTCTTTGGTAATCTTATGGATGCTGGAAATCTGTCGATT       1299
                   *********************************************************


pPGM            TGCGGGGAAGAGAGTTTTGGAACAGGTTCTGACCACATTCGTGAGAAAGACGGAATCTGG       3600
6G70620         TGCGGGGAAGAGAGTTTTGGAACAGGTTCTGACCACATTCGTGAGAAAGACGGAATCTG-       1357
                ***********************************************************
                Intron 16
PGM             TAACTTTCTTATTTTTGTATTGAGAATAGCGGGTCGAGCATTTATCAAACATTATCTAAG       3660
6G70620         ------------------------------------------------------------       1357

                              Exon 17
pPGM            TTTCTCCGACTTATTAATATTATAGGGCTGTATTAGCTTGGCTTTCGATTATTGCTCACC       3720
6G70620         -------------------------GGCTGTATTAGCTTGGCTTTCGATTATTGCTCACC       1393
                                         ***********************************
                                 rug3-e
pPGM            GCAACAAAGACACGAAACCAGGGGAGAAATTGGTCTCTGTGTCTGATGTTGTGAAGGAGC       3780
6G70620         GCAACAAAGACACGAAACCAGGGGAGAAATTGGTCTCTGTGTCTGATGTTGTGAAGGAGC       1453
                ************************************************************
                                                Intron 17
pPGM            ATTGGGCAACCTATGGTAGAAATTTCTTTTCTAGATACGATTACGAGGTTGGTATCGATG       3840
6G70620         ATTGGGCAACCTATGGTAGAAATTTCTTTTCTAGATACGATTACGAG-------------       1501
                ***********************************************

pPGM            CTGCAATTGAAGTTTTATTTGTTGTATCACACACTTTGAAGTTTTATTTTTCTTTTGATT       3900
6G70620         ------------------------------------------------------------       1501

                                      Exon 18
pPGM            TTTGACAAATATAAATATAGGAATGTGAATCTGAAGGCGCGAATAAGATGATAGAGTACC       3960
```

```
6G70620        --------------------GAATGTGAATCTGAAGGCGCGAATAAGATGATAGAGTACC        1540
                                   ****************************************
                                                            Intron 18
pPGM           TACGAGAGCTTTTGTCTAAGAGCAAGCCTGGTGATAAGTATGGTAAGTTACTCACAACCA        4020
6G70620        TACGAGAGCTTTTGTCTAAGAGCAAGCCTGGTGATAAGTATG------------------        1581
               ******************************************

pPGM           CTTTCTTATCACAGACACGGAGACACGGACACCAAACACGACACTGACACTGACACTGAC        4080
6G70620        ------------------------------------------------------------        1581


pPGM           ACGTAATCATTAGCATACACTTCCCCTGAGTATATTTAAAGTGTGATGAGTTTTCTTGTA        4140
6G70620        ------------------------------------------------------------        1581

                       Exon 19                                 Intron 19
pPGM           CAGGAAGTTACGTCCTCCAGTTTGCCGATGATTTTACATACACTGATCCTGTAAGTTCTT        4200
6G70620        ---GAAGTTACGTCCTCCAGTTTGCCGATGATTTTACATACACTGATCCT----------        1631
                  ***********************************************

pPGM           ACAATATTCACATTCTTATCATGTTCATTTTTGTTTCTTCAACCTACGGTAAAGCAACCA        4260
6G70620        ------------------------------------------------------------        1631

                                                     Exon 20
pPGM           ACCATAGTTCAAATTCTGATTGAATATAAATATGCAGGTAGATGGAAGTGTAGTATCAAA        4320
6G70620        -------------------------------------GTAGATGGAAGTGTAGTATCAAA        1652
                                                    ***********************
                                                            rug3-b Intron 20
pPGM           ACAAGGGGTTCGGTTTGTTTTCACCGATGGTTCAAGAATTATTTATCGTTTATCAGTAAG        4380
6G70620        ACAAGGGGTTCGGTTTGTTTTCACCGATGGTTCAAGAATTATTTATCGTTTATCA-----        1705
               *******************************************************

pPGM           TGACGTCTGTTTAATTACTTACCCGAAAATTTATTAAATTAAATATTAAGTGATTACTTA        4440
6G70620        ------------------------------------------------------------        1705

                                      Exon 21
pPGM           CGGTGTTTTTGTTTACAGGGAACGGGTTCTGCTGGTGCAACTGTTAGAGTGTATATCGAA        4500
6G70620        ------------------GGAACGGGTTCTGCTGGTGCAACTGTTAGAGTGTATATCGAA        1749
                                 ******************************************

pPGM           CAGTTTGAACCAGATGTTTCTAAACACGACGTCGATGCTCAAATTGCCTTGAAACCATTA        4560
6G70620        CAGTTTGAACCAGATGTTTCTAAACACGACGTCGATGCTCAAATTGCCTTGAAACCATTA        1809
               ************************************************************
                       Intron 21
pPGM           ATAGGTTAATTTCATGTTCCGAAACGAATGTAATACTTTAATAAACTAGTCGATCTTTTC        4620
6G70620        ATAG--------------------------------------------------------        1813
               ****
                                                     Exon 22
pPGM           TTTTTGTATGATTGATTGATCTTACTATTATGCAGATTTAGCATTATCTGTTTCAAAGCT        4680
6G70620        -----------------------------------ATTTAGCATTATCTGTTTCAAAGCT        1838
                                                  *************************

pPGM           CAAAGACTTCACAGGGAGAGAGAAGCCTACAGTCATCACTTAATATAAGTTTGGTTTTTC        4740
6G70620        CAAAGACTTCACAGGGAGAGAGAAGCCTACAGTCATCACTTAA-----------------        1881
               *******************************************
```

Mutant	F primer for screening	R primer for screening
rug3-a	CAGACTCGACAACTTCATCATCTC	AGGGTGTTTCCGTAAATCTTG
rug3-b	TACGTCCTCCAGTTTGCC	AAGGCAATTTGAGCATCG
rug3-c	TATTGTCAACGCCAGCC	AGGGTGTTTCCGTAAATCTTG
rug3-d	CAGACTCGACAACTTCATCATCTC	AGGGTGTTTCCGTAAATCTTG
rug3-e	GATGCTGGAAATCTGTCG	ATCTTATTCGCGCCTTCAG

C. Mutations at *rug4:* sucrose synthase 1 (SUS1)
chr4:469235623-469239739

```
        Exon 1
SUS1     ATGGCTACTGATCGATTGACTCGTGTTCATAGTCTCCGAGAGAGGCTTGATGAAACCTTG    60
4g74860  ATGGCTACTGATCGATTGACTCGTGTTCATAGTCTCCGAGAGAGGCTTGATGAAACCTTG    60
         ************************************************************
                                                    Intron 1
SUS1     ACTGCTAATAGGAATGAAATTTTAGCTCTTCTTTCAAGGTAAGAGTTAATCTATCGTCAT    120
4g74860  ACTGCTAATAGGAATGAAATTTTAGCTCTTCTTTCAAG----------------------    98
         **************************************

SUS1     TTAATTTTGTTTATTACTATTTGAGTTCAATTTTGTTGAAAATGTTTGATGAAACTTTGA    180
4g74860  ------------------------------------------------------------    98

              Exon 2
SUS1     AGGATTGAAGCAAAGGGAAAGGGAATTTTGCAACACCATCAAGTGATTGCTGAGTTTGAA    240
4g74860  --GATTGAAGCAAAGGGAAAGGGAATTTTGCAACACCATCAAGTGATTGCTGAGTTTGAA    156
           **********************************************************

SUS1     GAAATTCCTGAAGAGAATAGACAGAAGCTGACTGATGGTGCATTTGGTGAAGTTCTCAGA    300
4g74860  GAAATTCCTGAAGAGAATAGACAGAAGCTGACTGATGGTGCATTTGGTGAAGTTCTCAGA    216
         ************************************************************
                Intron 2
SUS1     TCCACACAGGTATAATTTATTTTGATGATGAAGCACAGATATCCTGAACACGACACTGAC    360
4g74860  TCCACACAG---------------------------------------------------    227
         *********

SUS1     ACGATTTTTTCAGACGTGTCGGTACTACATAGATTTTGATCAATGATTGTTTTTGATTTT    420
4g74860  ------------------------------------------------------------    227

                                   Exon 3
SUS1     TTGATGATTTTCTAGGAAGCTATAGTTTTGCCACCATGGGTTGCTCTTGCTGTTCGTCCA    480
4g74860  ---------------GAAGCTATAGTTTTGCCACCATGGGTTGCTCTTGCTGTTCGTCCA    270
                        *********************************************

SUS1     AGGCCAGGTGTCTGGGAGTATCTGAGAGTGAATGTGCATGCTCTTGTGGTTGAAAATTTG    540
4g74860  AGGCCAGGTGTCTGGGAGTATCTGAGAGTGAATGTGCATGCTCTTGTGGTTGAAAATTTG    330
         ************************************************************
                                                    Intron 3
SUS1     CAACCTGCTGAGTTTCTCAAATTCAAGGAAGAACTTGTTGATGGAAGGTACTGATTAACA    600
4g74860  CAACCTGCTGAGTTTCTCAAATTCAAGGAAGAACTTGTTGATGGAAG-------------    375
         ***********************************************

SUS1     AATTGATTCAGTTTTTATAATCATTGGTTTTGTGATTTTGTTAATGTTTTTACACAAATT    660
4g74860  ------------------------------------------------------------    375

                                   Exon 4
SUS1     TCACTTTTGTTGCAGTGCTAATGGTAACTTTGTGCTTGAATTGGACTTTGAACCATTTAC    720
4g74860  ---------------TGCTAATGGTAACTTTGTGCTTGAATTGGACTTTGAACCATTTAC    422
                        *********************************************

SUS1     TGCATCTTTCCCTCGTCCTACTCTCAACAAATCAATTGGAAATGGTGTTCAGTTTCTCAA    780
4g74860  TGCATCTTTCCCTCGTCCTACTCTCAACAAATCAATTGGAAATGGTGTTCAGTTTCTCAA    482
         ************************************************************
                    rug4-a
SUS1     CCGTCACCTTTCTGCCAAACTCTTCCATGACAAGGAGAGTTTGCATCCACTTTTGGAATT    840
4g74860  CCGTCACCTTTCTGCCAAACTCTTCCATGACAAGGAGAGTTTGCATCCACTTTTGGAATT    542
         ************************************************************
                                              Intron 4
SUS1     TCTCAGACTTCACAGCTACAAGGGGAAGGTAACAACTCGCTACTTCCATGATCTTGCATG    900
4g74860  TCTCAGACTTCACAGCTACAAGGGGAAG--------------------------------    570
         ****************************
                                                      Exon 5
SUS1     CTTTTGTGTCATTCAAATTATTGATCTTGTGTGTCTTTTGATTTGCAGACATTGATGTTG    960
4g74860  ------------------------------------------------ACATTGATGTTG    582
                                                         ************
```

```
SUS1      AATGACAGAATTCAGAACCCTGATTCTCTTCAACATGTTCTGAGAAAGGCTGAAGAGTAT     1020
4g74860   AATGACAGAATTCAGAACCCTGATTCTCTTCAACATGTTCTGAGAAAGGCTGAAGAGTAT     642
          ************************************************************

SUS1      CTAGGCACAGTTGCTCCGGATACACCGTACTCCGAATTCGAACACAGGTTCCAGGAGATT     1080
4g74860   CTAGGCACAGTTGCTCCGGATACACCGTACTCCGAATTCGAACACAGGTTCCAGGAGATT     702
          ************************************************************

SUS1      GGTTTGGAGAGAGGTTGGGGAGACACCGCAGAGCGCGTGCTCGAGTCCATTCAGCTTCTA     1140
4g74860   GGTTTGGAGAGAGGTTGGGGAGACACCGCAGAGCGCGTGCTCGAGTCCATTCAGCTTCTA     762
          ************************************************************

SUS1      CTGGATCTTCTTGAGGCTCCTGATCCTTGCACTCTTGAGACTTTCCTTGACAGAATCCCT     1200
4g74860   CTGGATCTTCTTGAGGCTCCTGATCCTTGCACTCTTGAGACTTTCCTTGACAGAATCCCT     822
          ************************************************************

SUS1      ATGGTGTTTAATGTTGTTATTCTTTCTCCTCATGGTTACTTTGCTCAAGATGATGTCTTG     1260
4g74860   ATGGTGTTTAATGTTGTTATTCTTTCTCCTCATGGTTACTTTGCTCAAGATGATGTCTTG     882
          ************************************************************
                                     Intron 5
SUS1      GGATACCCTGATACCGGTGGTCAGGTTGGTTTATAAGATCGAATGTTTCTGTTGTTTGTG     1320
4g74860   GGATACCCTGATACCGGTGGTCAG------------------------------------     910
          ************************

SUS1      AATAATCTCATACTGTTTTTATACCTAACTCACATGCTTTACTTGATCTTCAATCTAGGT     1380
4g74860   ----------------------------------------------------------GT     910
                                                                    **
           Exon 6
SUS1      TGTTTACATTTTGGATCAAGTTCGTGCCTTGGAGAGCGAGATGCTCAATCGCATTAAGAA     1440
4g74860   TGTTTACATTTTGGATCAAGTTCGTGCCTTGGAGAGCGAGATGCTCAATCGCATTAAGAA     968
          ************************************************************
                                             Intron 6
SUS1      ACAAGGCTTGGATATCGTTCCTCGCATTCTCATTGTAAGTTCCGGTTTATTTTATTCGGT     1500
4g74860   ACAAGGCTTGGATATCGTTCCTCGCATTCTCATT--------------------------     1002
          **********************************

SUS1      AACTTGTAAGTCATAGTTCGTTGTATATTGCGTACGGGCTAATTCTCTAATTGGTGCTTG     1560
4g74860   ------------------------------------------------------------     1002

                                                    Exon 7
SUS1      TGGTTGTGGATGATGTTTCTGTTGAATACAGATCACTCGTCTTCTCCCAGACGCAGTCGG     1620
4g74860   -------------------------------ATCACTCGTCTTCTCCCAGACGCAGTCGG     1031
                                         *****************************

SUS1      AACTACTTGTGGCCAACGACTCGAGAAGGTCTATGGAACCGAGCATTGCCACATTCTTCG     1680
4g74860   AACTACTTGTGGCCAACGACTCGAGAAGGTCTATGGAACCGAGCATTGCCACATTCTTCG     1091
          ************************************************************

SUS1      AGTTCCCTTCAGAGATCAGAAGGGAATTGTTCGCAAGTGGATCTCGCGTTTCGAAGTCTG     1740
4g74860   AGTTCCCTTCAGAGATCAGAAGGGAATTGTTCGCAAGTGGATCTCGCGTTTCGAAGTCTG     1151
          ************************************************************
                                             Intron 7
SUS1      GCCATATCTAGAAACCTACACCGAGGTACCACTTCGATCCATAATCTTGACACTTTTCAA     1800
4g74860   GCCATATCTAGAAACCTACACCGAG-----------------------------------     1177
          *************************
                                                            Exon 8
SUS1      ACATTTTGATGTTTCTTTTATTTTTAATTATATGTATCTTGTCGTTAGGATGTTGCTCAT     1860
4g74860   ------------------------------------------------GATGTTGCTCAT     1188
                                                          ************

SUS1      GAGCTTGCCAAAGAGTTGCAAGGCAAACCAGATCTGATTGTTGGAAACTACAGTGATGGA     1920
4g74860   GAGCTTGCCAAAGAGTTGCAAGGCAAACCAGATCTGATTGTTGGAAACTACAGTGATGGA     1248
          ************************************************************
                                                    rug4-c Intron 8
SUS1      AACATTGTTGCTTCTTTGTTGGCACATAAATTAGGTGTCACTCAGGTTTGTATTCATCAA     1980
4g74860   AACATTGTTGCTTCTTTGTTGGCACATAAATTAGGTGTCACTCAG---------------     1292
          *********************************************

SUS1      CACTTACCAGTTGTAAACTGTAACATTCTGTTAACTATGCTTTGTTCTAATGTTTTTCCT     2040
4g74860   ------------------------------------------------------------     1292
```

```
                                   Exon 9
SUS1        TGTTATTACAGTGTACTATTGCTCATGCACTTGAGAAGACTAAGTATCCTGAATCTGATA     2100
4g74860     -----------TGTACTATTGCTCATGCACTTGAGAAGACTAAGTATCCTGAATCTGATA     1342
                       *************************************************

SUS1        TTTACTGGAAAAAATTCGAAGAGAAGTATCACTTCTCCTGCCAATTTACCGCCGATCTTT     2160
4g74860     TTTACTGGAAAAAATTCGAAGAGAAGTATCACTTCTCCTGCCAATTTACCGCCGATCTTT     1402
            ************************************************************

SUS1        TCGCAATGAACCACACAGATTTCATCATCACAAGTACCTTCCAAGAGATTGCTGGAAGGT     2220
4g74860     TCGCAATGAACCACACAGATTTCATCATCACAAGTACCTTCCAAGAGATTGCTGGAAG--     1459
            **********************************************************
             Intron 9
SUS1        GTGATATGATATCCTTTTGTTTGCAATTTTTGTTTCTTTGATTATCAGGCTTACTGAAAT     2280
4g74860     ------------------------------------------------------------     1459

                                                  Exon 10
SUS1        TGTTGTGTTTTCTTGTGCAGCAAGGATACTGTTGGACAGTATGAGAGTCACACTGCCTTC     2340
4g74860     --------------------CAAGGATACTGTTGGACAGTATGAGAGTCACACTGCCTTC     1500
                                ****************************************

SUS1        ACTCTTCCGGGACTGTACCGTGTCGTGCACGGTATTGATGTCTTTGATCCTAAGTTCAAC     2400
4g74860     ACTCTTCCGGGACTGTACCGTGTCGTGCACGGTATTGATGTCTTTGATCCTAAGTTCAAC     1560
            ************************************************************

SUS1        ATTGTATCTCCTGGAGCTGATCAGACCATTTACTTCCCTTACACCGAAACTAGCCGCAGG     2460
4g74860     ATTGTATCTCCTGGAGCTGATCAGACCATTTACTTCCCTTACACCGAAACTAGCCGCAGG     1620
            ************************************************************

SUS1        TTGACATCATTCTACCCTGAAATTGAAGAGCTTCTTTACAGCACAGTGGAAAATGAAGAA     2520
4g74860     TTGACATCATTCTACCCTGAAATTGAAGAGCTTCTTTACAGCACAGTGGAAAATGAAGAA     1680
            ************************************************************
                        Intron 10
SUS1        CACATGTGAGATTCTTTCATTCTCGTTTTATGATAATGTTATGTTGTTGGCATTGTCGCA     2580
4g74860     CACAT-------------------------------------------------------     1684
            *****
                                                                 Exon 11
SUS1        TGAAAGTTTTTTCTAATCAAATGCGACTCCTTTGTGACAGATGTGTGCTCAAGGACCGCA     2640
4g74860     ----------------------------------------ATGTGTGCTCAAGGACCGCA     1705
                                                    ********************
                                       rug4-b
SUS1        GCAAGCCGATTATCTTCACCATGGCAAGGTTGGACCGTGTGAAGAACATTACAGGACTTG     2700
4g74860     GCAAGCCGATTATCTTCACCATGGCAAGGTTGGACCGTGTGAAGAACATTACAGGACTTG     1765
            ************************************************************

SUS1        TTGAATGGTACGGAAAGAATGCCAAGCTTCGTGAGTTGGTGAACCTCGTAGTTGTTGCCG     2760
4g74860     TTGAATGGTACGGAAAGAATGCCAAGCTTCGTGAGTTGGTGAACCTCGTAGTTGTTGCCG     1825
            ************************************************************

SUS1        GAGACAGAAGGAAGGAGTCAAAGGACTTGGAAGAGAAAGCTGAGATGAAGAAGATGTACG     2820
4g74860     GAGACAGAAGGAAGGAGTCAAAGGACTTGGAAGAGAAAGCTGAGATGAAGAAGATGTACG     1885
            ************************************************************

SUS1        AACATATCGAGACCTACAAGTTGAATGGCCAATTCAGATGGATTTCGTCTCAGATGAACC     2880
4g74860     AACATATCGAGACCTACAAGTTGAATGGCCAATTCAGATGGATTTCGTCTCAGATGAACC     1945
            ************************************************************

SUS1        GTGTCAGAAACGGAGAGCTCTACCGTGTGATCTGTGACACAAAAGGAGCTTTCGTGCAGC     2940
4g74860     GTGTCAGAAACGGAGAGCTCTACCGTGTGATCTGTGACACAAAAGGAGCTTTCGTGCAGC     2005
            ************************************************************

SUS1        CTGCTGTCTACGAGGCTTTCGGTCTAACAGTCGTTGAGGCCATGGCTACCGGATTACCAA     3000
4g74860     CTGCTGTCTACGAGGCTTTCGGTCTAACAGTCGTTGAGGCCATGGCTACCGGATTACCAA     2065
            ************************************************************

SUS1        CATTCGCAACACTCAATGGTGGACCTGCTGAGATCATTGTCCATGGAAAATCTGGATTCC     3060
4g74860     CATTCGCAACACTCAATGGTGGACCTGCTGAGATCATTGTCCATGGAAAATCTGGATTCC     2125
            ************************************************************

SUS1        ACATCGATCCATACCACGGCGACCGCGCTGCTGATCTCCTAGTCGAATTCTTCGAGAAAG     3120
4g74860     ACATCGATCCATACCACGGCGACCGCGCTGCTGATCTCCTAGTCGAATTCTTCGAGAAAG     2185
```

```
          ************************************************************

SUS1      TTAAGACTGATCCATCTCACTGGGACAAGATCTCTCAAGGTGGTCTCCAACGTATTGAAG     3180
4g74860   TTAAGACTGATCCATCTCACTGGGACAAGATCTCTCAAGGTGGTCTCCAACGTATTGAAG     2245
          ************************************************************
                       Intron 11
SUS1      AGAAGTAAGCAAGCTCCTATAACCAAACCAACCATGTTACTATTACATAGGTGTCTTTGT     3240
4g74860   AGAA--------------------------------------------------------     2250
          ****

SUS1      CTTTGTTTGGTTGTGTGGTTGTGTGAATTATGATTAACATGTTTTCTTTTCTGGTTTTGT     3300
4g74860   ------------------------------------------------------------     2250

                              Exon 12
SUS1      TCCAAAGGTACACATGGCAAATTTACTCTCAGAGGCTTCTTACACTCACTGGTGTCTATG     3360
4g74860   -------GTACACATGGCAAATTTACTCTCAGAGGCTTCTTACACTCACTGGTGTCTATG     2302
                 *****************************************************

SUS1      GTTTCTGGAAACATGTTTCTAACCTCGACCGCCTCGAGAGCCGCCGCTATCTCGAGATGT     3420
4g74860   GTTTCTGGAAACATGTTTCTAACCTCGACCGCCTCGAGAGCCGCCGCTATCTCGAGATGT     2362
          ************************************************************
                                                   Intron 12
SUS1      TCTATGCTCTCAAGTACCGCAAGTTGGTAAGTTCTATAGGCACACACATACTATATAACA     3480
4g74860   TCTATGCTCTCAAGTACCGCAAGTTG------------------------------         2392
          **************************

SUS1      TCTGATCAATGTTTTTCTATGAAGTTACTAATGAAATATGTTACATTTTCTTTGTTCCTC     3540
4g74860   ------------------------------------------------------------     2392

                       Exon 13
SUS1      AGGCTGAGTCTGTGCCTCTAGCTGTTGAGGAGTGATTGATGAAATGGAGGAACCGGCTTT     3600
4g74860   --GCTGAGTCTGTGCCTCTAGCTGTTGAGGAGTGA-------------------------     2421
            *********************************
```

Mutant	F primer for screening	R primer for screening
rug4-a	TGGTTGAAAATTTGCAACC	TAGCTGTGAAGTCTGAGAAATTCC
rug4-b	TTACACCGAAACTAGCCGC	ATTCAACTTGTAGGTCTCGATATGTTC
rug4-c	CTTGAGACTTTCCTTGACAGAATCCC	CAGGATACTTAGTCTTCTCAAGTGC

D. Mutations at *rug5:* starch synthase 2 (SS2)
chr1:339411495-339415334

```
             Exon 1
SS2          ATGATGCTATCACTAGGTTCTGATGCAACTGTGTTACCTTTTCATGCTAAGAATCTCAAA  60
1g53180      ATGATGCTATCACTAGGTTCTGATGCAACTGTGTTACCTTTTCATGCTAAGAATCTCAAA  60
             ************************************************************

SS2          TTTACTCCAAAGTTAAGTACTTTGAATGGTGATTTAGCATTTAGTAAGGGTTTGGGTGTT  120
1g53180      TTTACTCCAAAGTTAAGTACTTTGAATGGTGATTTAGCATTTAGTAAGGGTTTGGGTGTT  120
             ************************************************************

SS2          GGTAGATTGAACTGTGGAAGTGTTAGATTGAATCATAAGCAGCATGTAAGAGCTGTTGGT  180
1g53180      GGTAGATTGAACTGTGGAAGTGTTAGATTGAATCATAAGCAGCATGTAAGAGCTGTTGGT  180
             ************************************************************

SS2          AAGAGTTTTGGTGCAGATGAGAATGGAGATGGGTCAGAGGATGATGTTGTTAATGCTACA  240
1g53180      AAGAGTTTTGGTGCAGATGAGAATGGAGATGGGTCAGAGGATGATGTTGTTAATGCTACA  240
             ************************************************************
                                                           Intron 1
SS2          ATTGAAAAGAGCAAAAAGGTTCTTGCTTTGCAAAGGGAACTTATTCAACAGGTATTGTGA  300
1g53180      ATTGAAAAGAGCAAAAAGGTTCTTGCTTTGCAAAGGGAACTTATTCAACAG---------  291
             ***************************************************

SS2          AAATTTTGATTTTCTAAAAGGGTTGTGATTTTCATCTTTAGGTCTAAAGGTTTTTTGGTT  360
```

```
1g53180     ------------------------------------------------------------  291

SS2         GAGACCCTTTAAGCTATTAAGTTGATTGATTCTGTTTCTCTTTTTTTTTGTGATGATTTT  420
1g53180     ------------------------------------------------------------  291

SS2         TACTTGTTGGTCATTTGTTGAAATTGGAACAAATTAGGACTTTATTAGAGAGATGATTGA  480
1g53180     ------------------------------------------------------------  291

SS2         TTTTTCTCTCGATGATTCTAAATTGATTATGAATGTGTAGAATTGATTCTGACATGACAT  540
1g53180     ------------------------------------------------------------  291

SS2         AGTTGTTCTCGAGTAGAATTGATTATGCTTTTTCAGAATTGATTATACCTGAAGCTTGAA  600
1g53180     ------------------------------------------------------------  291

SS2         TTTGTAGCTTTTGAGTCTAAATGTGAATTTTACACTGAAATATTTAGTTCGAATCATTTT  660
1g53180     ------------------------------------------------------------  291

SS2         TACAAGAATTTATTCAAACATAAATCACTTTACGTTTAACTCACTTTTAGCCAGAATCAC  720
1g53180     ------------------------------------------------------------  291

SS2         TTTAACATAATCTATTCACTCAAAATCAATTTTCTTCACAGCAATACCAAACACACACTA  780
1g53180     ------------------------------------------------------------  291

SS2         AAGCAATGTCTAATATGTATACTTCTCCTCACTCATGAAGTAGCAATGACAATTTTTTAA  840
1g53180     ------------------------------------------------------------  291

SS2         CCAATGTTGTCAAATAGCGGCACTATAGTTTAGCAGAATTTGAATAAAATGCTATTGTAT  900
1g53180     ------------------------------------------------------------  291

SS2         TGATCTGTTTGCGAATTTAGAGTTGAATGCAAAACTATATTGCAGAGTTTTATTGTTGGC  960
1g53180     ------------------------------------------------------------  291

SS2         AATATCTGCCTCTTAGAGTTTCTATTTTAGTCTCTAGTCGTGAGGTTCTACGGCTATCGT  1020
1g53180     ------------------------------------------------------------  291

SS2         TTATGCCACTAGGTAAACTTAAATAAGTCAATCCAAACGAGTCGTTGGTCTTTCCGACTC  1080
1g53180     ------------------------------------------------------------  291

SS2         TCCCTAGTCCCTACATTCTTTCATAAGCAGTTAACCCTTACGGTTATTATAAACATTCAT  1140
1g53180     ------------------------------------------------------------  291

                                                    Exon 2
SS2         GTGGTTATTTTATTTATCTTCTTTTTTTGATATTGTAGATTGCTGAAAGAAAGAAACTAG  1200
1g53180     --------------------------------------ATTGCTGAAAGAAAGAAACTAG  313
                                                  **********************

SS2         TTTCTTCTATAGATAGTGACAGCATTCCTGGATTGGAAGGAAACGGTGTTTCTTATGAAA  1260
1g53180     TTTCTTCTATAGATAGTGACAGCATTCCTGGATTGGAAGGAAACGGTGTTTCTTATGAAA  373
            ************************************************************

SS2         GCGGTGAAAAATCTCTGTCAAGGGACTCAAATCCACAGAAAGGTTCTTCCAGCAGCGGCA  1320
1g53180     GCGGTGAAAAATCTCTGTCAAGGGACTCAAATCCACAGAAAGGTTCTTCCAGCAGCGGCA  433
            ************************************************************

SS2         GTGCTGTTGAAAACCAAAACGGTGGCACTGTTTTCAGCAACTATGTCCGTTCTAAAGAAA  1380
1g53180     GTGCTGTTGAAAACCAAAACGGTGGCACTGTTTTCAGCAACTATGTCCGTTCTAAAGAAA  493
            ************************************************************
```

```
SS2          CGGAGACATGGGCCGTTTCATCTGTTGGCATTAATCAAGGTTTTGATGAAATTGAAAAGA  1440
1g53180      CGGAGACATGGGCCGTTTCATCTGTTGGCATTAATCAAGGTTTTGATGAAATTGAAAAGA  553
             ************************************************************

SS2          AAAACGACGCTGTAAAGGCGTCGTCTAAGCTACACTTCAATGAACAAATTAAAAACAAGC  1500
1g53180      AAAACGACGCTGTAAAGGCGTCGTCTAAGCTACACTTCAATGAACAAATTAAAAACAAGC  613
             ************************************************************

SS2          TGTACGAGAGACCTGATACGAAAGACATTTCAAGTAGCATCAGAACTTCTAGTCTAAAGT  1560
1g53180      TGTACGAGAGACCTGATACGAAAGACATTTCAAGTAGCATCAGAACTTCTAGTCTAAAGT  673
             ************************************************************

SS2          TTGAAAATTTCGAAGGTGCGAATGAACCAAGTTCAAAGGAGGTTGCTAATGAAGCTGAAA  1620
1g53180      TTGAAAATTTCGAAGGTGCGAATGAACCAAGTTCAAAGGAGGTTGCTAATGAAGCTGAAA  733
             ************************************************************

SS2          ATTTTGAGAGCGGCGGTGAAAAGCCACCACCGTTGGCTGGGACCAATGTTATGAACATTA  1680
1g53180      ATTTTGAGAGCGGCGGTGAAAAGCCACCACCGTTGGCTGGGACCAATGTTATGAACATTA  793
             ************************************************************
                                                       Intron 2
SS2          TATTGGTTTCTGCAGAATGCGCTCCTTGGTCGAAAACAGGTAACTTAACATTATTTCATA  1740
1g53180      TATTGGTTTCTGCAGAATGCGCTCCTTGGTCGAAAACAG---------------------  830
             ***************************************

SS2          GAAGAGTTTTCCTTGATAAATGCATGTTGCTGAGGTTCTCTATAATAAACCAAGATATTC  1800
1g53180      ------------------------------------------------------------  830

SS2          ACCGGTAAGAGAGAACACGTTCAAATATTCGCAGACGAAGTCTTCAATCTTTTCTGTCCT  1860
1g53180      ------------------------------------------------------------  830

                                                                  Exon 3
SS2          TCCATGTATGCTGCATTTTTTTTCTATATAATTTACGGTTTTAATATGAACGAATAGGCG  1920
1g53180      ---------------------------------------------------------GCG  835
                                                                      ***

               rug5-b
SS2          GGCTTGGAGATGTTGCTGGATCACTCCCGAAGGCTTTGGCTCGGCGTGGACATAGAGTTA  1980
1g53180      GGCTTGGAGATGTTGCTGGATCACTCCCGAAGGCTTTGGCTCGGCGTGGACATAGAGTTA  895
             ************************************************************
               Intron 3
SS2          TGGTATAACGCTATTCCCTTCATGTCTCATTACAAGATAAGATACTTCGATAAATTTTCA  2040
1g53180      TG----------------------------------------------------------  896
             **

SS2          CTCTCCTTTCATTATGAACTGTTTCCGTTTTCTTATTTCTTTTCTTTATTTTCGTTATCT  2100
1g53180      ------------------------------------------------------------  896

                     Exon 4
SS2          TCTTATAGATTGTTGCACCTCATTATGGTAATTATGCTGAAGCACACGATATAGGAGTAC  2160
1g53180      --------ATTGTTGCACCTCATTATGGTAATTATGCTGAAGCACACGATATAGGAGTAC  949
                     ****************************************************
                                        Intron 4
SS2          GGAAGAGATACAAAGTAGCTGGTCAGGTGTGCCGCGAGCGTCTACTCTATTCCTAATTTA  2220
1g53180      GGAAGAGATACAAAGTAGCTGGTCAG----------------------------------  974
             **************************

SS2          TGATTCCTCGAAACAGTTATTTTCTACCACTTTGGTGTAGTAATGCTTCAAAGTTTTCAC  2280
1g53180      ------------------------------------------------------------  683

                     Exon 5
SS2          TTTACAGGACATGGAAGTGACGTATTTCCATACTTATATTGATGGTGTCGATATTGTTTT  2340
1g53180      -------GACATGGAAGTGACGTATTTCCATACTTATATTGATGGTGTCGATATTGTTTT  1028
                    *****************************************************

SS2          TATTGACAGTCCAATATTTCGCAATCTAGAGAGTAATATATATGGCGGAAACCGATTGGT  2400
1g53180      TATTGACAGTCCAATATTTCGCAATCTAGAGAGTAATATATATGGCGGAAACCGATTG--  1087
             **********************************************************
               Intron 5
SS2          AATTTTTGATTGGAATGGATCTTGCACTAGTATCAGTTACCCTTACTATCTCAGTCTTTT  2460
1g53180      ------------------------------------------------------------  1087
```

```
                                              Exon 6
SS2          GATTTTTGTTGTCCTTTAATGGAACAGGATATTCTAAGGCGCATGGTGTTGTTTTGCAAG  2520
1g53180      ---------------------------GATATTCTAAGGCGCATGGTGTTGTTTTGCAAG  1119
                                        *********************************
                               Intron 6
SS2          GCGGCGGTTGAGGTACACGAACTAAATTGTTTTATGATTATAACTTGTTTGAATTGAATC  2580
1g53180      GCGGCGGTTGAG------------------------------------------------  1131
             ************
                                                                  Exon 7
SS2          CATTTATTTGAGCTTATCTACTAGCGCACGCATATTGATTATTTATTCGTTAGGTTCCTT  2640
1g53180      -----------------------------------------------------GTTCCTT  1138
                                                                  *******

SS2          GGCATGTTCCGTGTGGTGGAATTTGCTATGGAGATGGAAATCTGGTCTTCATAGCAAATG  2700
1g53180      GGCATGTTCCGTGTGGTGGAATTTGCTATGGAGATGGAAATCTGGTCTTCATAGCAAATG  1198
             ************************************************************

SS2          ATTGGCATACTGCATTGCTGCCGGTGTATCTGAAAGCATATTATCGCGATCATGGTTTGA  2760
1g53180      ATTGGCATACTGCATTGCTGCCGGTGTATCTGAAAGCATATTATCGCGATCATGGTTTGA  1258
             ************************************************************
                                                           Intron 7
SS2          TGAACTATACACGATCGGTTCTCGTGATTCATAACATAGCTCACCAGGTTCGTTCTGAAT  2820
1g53180      TGAACTATACACGATCGGTTCTCGTGATTCATAACATAGCTCACCAG-------------  1304
             ***********************************************

SS2          CTTCGTATATGTTGCCTTTGTTGTTATGCACGTATACACACTGACACACGTTTACTCCTG  2880
1g53180      ------------------------------------------------------------  1013

               Exon 8
SS2          CAGGGTCGGGGCCCGGTCGAGGATTTCAACACCGTGGATTTATCTGGAAACTACTTAGAC  2940
1g53180      ---GGTCGGGGCCCGGTCGAGGATTTCAACACCGTGGATTTATCTGGAAACTACTTAGAC  1362
                ********************************************************* 

SS2          CTTTTCAAAATGTACGACCCTGTTGGAGGTGAGCACTTCAACATCTTTGCAGCCGGTTTA  3000
1g53180      CTTTTCAAAATGTACGACCCTGTTGGAGGTGAGCACTTCAACATCTTTGCAGCCGGTTTA  1422
             ************************************************************

SS2          AAAACCGCCGACAGGATTGTCACCGTCAGTCACGGTTACGCATGGGAGCTTAAAACTTCA  3060
1g53180      AAAACCGCCGACAGGATTGTCACCGTCAGTCACGGTTACGCATGGGAGCTTAAAACTTCA  1482
             ************************************************************

SS2          GAAGGTGGTTGGGGTTTGCATAACATCATAAATGAAAGTGACTGGAAATTTCGGGGAATT  3120
1g53180      GAAGGTGGTTGGGGTTTGCATAACATCATAAATGAAAGTGACTGGAAATTTCGGGGAATT  1542
             ************************************************************

SS2          GTGAACGGTGTCGACACAAAAGATTGGAACCCTCAATTCGACGCGTACTTGACATCAGAC  3180
1g53180      GTGAACGGTGTCGACACAAAAGATTGGAACCCTCAATTCGACGCGTACTTGACATCAGAC  1602
             ************************************************************

SS2          GGCTACACTAACTACAACCTGAAAACCCTACAAACCGGCAAGCGTCAATGTAAGGCAGCT  3240
1g53180      GGCTACACTAACTACAACCTGAAAACCCTACAAACCGGCAAGCGTCAATGTAAGGCAGCT  1662
             ************************************************************

SS2          TTGCAAAGGGAGCTCGGTTTGCCTGTCCGTGAGGATGTTCCGATAATTTCCTTCATCGGA  3300
1g53180      TTGCAAAGGGAGCTCGGTTTGCCTGTCCGTGAGGATGTTCCGATAATTTCCTTCATCGGA  1722
             ************************************************************

SS2          AGGCTCGATCACCAGAAAGGTGTTGATCTTATAGCCGAAGCGATTCCTTGGATGATGAGC  3360
1g53180      AGGCTCGATCACCAGAAAGGTGTTGATCTTATAGCCGAAGCGATTCCTTGGATGATGAGC  1782
             ************************************************************

SS2          CACGATGTTCAACTAGTCATGTTGGGAACCGGAAGAGCTGATTTAGAACAAATGCTCAAA  3420
1g53180      CACGATGTTCAACTAGTCATGTTGGGAACCGGAAGAGCTGATTTAGAACAAATGCTCAAA  1842
             ************************************************************

SS2          GAATTCGAAGGCCAACACTGCGACAAAATCCGAAGTTGGGTTGGTTTTTCGGTTAAGATG  3480
1g53180      GAATTCGAAGGCCAACACTGCGACAAAATCCGAAGTTGGGTTGGTTTTTCGGTTAAGATG  1902
             ************************************************************

SS2          GCTCATAGGATAACAGCAGGTTCAGATATATTACTCATGCCGTCAAGATTCGAGCCGTGT  3540
1g53180      GCTCATAGGATAACAGCAGGTTCAGATATATTACTCATGCCGTCAAGATTCGAGCCGTGT  1962
```

```
                 ************************************************************

SS2              GGACTGAATCAACTCTATGCGATGAGTTACGGAACAGTTCCTGTTGTACACGGGGTGGGC  3600
1g53180          GGACTGAATCAACTCTATGCGATGAGTTACGGAACAGTTCCTGTTGTACACGGGGTGGGC  2022
                 ************************************************************
                                                                 rug5-c
SS2              GGACTCAGAGACACAGTGCAGCCTTTTAATCCGTTCGACGAATCGGGTGTCGGTTGGACA  3660
1g53180          GGACTCAGAGACACAGTGCAGCCTTTTAATCCGTTCGACGAATCGGGTGTCGGTTGGACA  2082
                 ************************************************************
                                                        rug5-a
SS2              TTTGATCGCGCCGAAGCTAACAAGTTAATGGCGGCATTATGGAATTGCTTATTGACTTAT  3720
1g53180          TTTGATCGCGCCGAAGCTAACAAGTTAATGGCGGCATTATGGAATTGCTTATTGACTTAT  2142
                 ************************************************************

SS2              AAGGATTATAAGAAGAGCTGGGAAGGGATTCAAGAAAGAGGGATGTCACAGGATCTTAGC  3780
1g53180          AAGGATTATAAGAAGAGCTGGGAAGGGATTCAAGAAAGAGGGATGTCACAGGATCTTAGC  2202
                 ************************************************************

SS2              TGGGACAATGCTGCTCAACAATATGAAGAGGTTCTTGTTGCTGCCAAATACCAATGGTGA  3840
1g53180          TGGGACAATGCTGCTCAACAATATGAAGAGGTTCTTGTTGCTGCCAAATACCAATGGTGA  2262
                 ************************************************************

SS2              TTTCTTGACATGCACTCCATCTTTGTAGTTTGATCAAAACGAAGGGCTTTCATGCTGAAT  3900
1g53180          ------------------------------------------------------------
```

Mutant	F primer for screening	R primer for screening
rug5-a	GGAACCGGAAGAGCTGATTT	GAAAGCCCTTCGTTTTGATCA
rug5-b	ACAAGCTGTACGAGAGACCT	CGTACTCCTATATCGTGTGCTTC
rug5-c	GGAACCGGAAGAGCTGATTT	GAAAGCCCTTCGTTTTGATCA

E. Mutations at *lam:* granule-bound starch synthase I (GBSS1) chr6:63031214-63034472

```
GBSS1            CGATCACTCTTCTCTCACCGCCGGTGTGTCATCAGCTACTATCTATAACTTCATCACACT      -120
GBSS1            CTTCTCCCAGATCCAGATTAATCACCTTAATAATATCACATTTATTTTACTAAAACTAAT       -60
GBSS1            TATTAAAACTAAGTTGATGAATTTGGTTTGATACATGCAGAAACAGATTTTGACACAAAA         0
                 Exon 1
GBSS1            ATGGCAACAATAACGGGATCTTCAATGCCGACGAGAACCGCGTGCTTCAATTACCAAGGA        60
6g20480          ATGGCAACAATAACGGGATCTTCAATGCCGACGAGAACCGCGTGCTTCAATTACCAAGGA        60
                 ************************************************************

GBSS1            AGATCAGCAGAGTCTAAACTGAATTTGCCTCAGATCACATTCAATAACAACCAAGCGTTT       120
6g20480          AGATCAGCAGAGTCTAAACTGAATTTGCCTCAGATCACATTCAATAACAACCAAGCGTTT       120
                 ************************************************************

GBSS1            ACGCATATTGGTTTGAGATCTCTCAACAAGCTGCACGTGCGTACCGCACGTGCTACTTCC       180
6g20480          ACGCATATTGGTTTGAGATCTCTCAACAAGCTGCACGTGCGTACCGCACGTGCTACTTCC       180
                 ************************************************************

GBSS1            GGTTCTTCAGATACCAGTGAAAAGTCATTGGGGAAAATAGTGTGTGGAATGAGCTTGGTG       240
6g20480          GGTTCTTCAGATACCAGTGAAAAGTCATTGGGGAAAATAGTGTGTGGAATGAGCTTGGTG       240
                 ************************************************************
                          lam-a
GBSS1            TTTGTTGGAGCTGAAGTTGGACCTTGGAGTAAAACTGGTGGACTCGGTGATGTTCTTGGT       300
6g20480          TTTGTTGGAGCTGAAGTTGGACCTTGGAGTAAAACTGGTGGACTCGGTGATGTTCTTGGT       300
                 ************************************************************
                                       Intron 1
GBSS1            GGTCTTCCACCTGTTTTAGCAGTAAGTTACTTTTGCATTACACTTATCATCCTCGTGGAT       360
6g20480          GGTCTTCCACCTGTTTTAGCA---------------------------------------       322
                 *********************
```

```
GBSS1           ACGATATCACATTGACATGTTGACACCTCGAGTAGTTTAGAAAAAATGAATGTAATTGAA        420
6g20480         ------------------------------------------------------------        322

                              lam-c Exon 2
GBSS1           TGCATGCAATTTTTGTGATAATTTTATTTTTTAGGGAAATGGACATCGAGTTATGACTGT        480
6g20480         -----------------------------------GGAAATGGACATCGAGTTATGACTGT        347
                                                   *************************
                                                     Intron 2
GBSS1           GTCACCGCGTTATGATCAATACAAGGATGCATGGGATACGAATGTGCTGGTTGAGGTAAA        540
6g20480         GTCACCGCGTTATGATCAATACAAGGATGCATGGGATACGAATGTGCTGGTTGAG-----        406
                *******************************************************

GBSS1           AAAAATTGTTTATGTATAAATCTTGAGAGTATTAAGCAAGAATTACGGTTATTAATGATT        600
6g20480         ------------------------------------------------------------        406

                              Exon 3
GBSS1           CGTTAATTTTTTGTTGTTGTTGTTGGTAGGTTAAAGTTGGAGATAAAATTGAAACTGTGC        660
6g20480         -----------------------------GTTAAAGTTGGAGATAAAATTGAAACTGTGC        433
                                             *******************************

GBSS1           GTTTCTTTCACTGTTACAAGAGAGGAGTTGATCGTGTTTTTGTGGATCATCCACTTTTCC        720
6g20480         GTTTCTTTCACTGTTACAAGAGAGGAGTTGATCGTGTTTTTGTGGATCATCCACTTTTCC        493
                ************************************************************
                        Intron 3
GBSS1           TTGAAAGGGTTTGATTTCTATGCTTTTGTTTTCATCAATCAGTGGAATGTTTAAAGATAA        780
6g20480         TTGAAAGG----------------------------------------------------        501
                ********
                                      Exon 4
GBSS1           ACTGATTTGTTTATTGATATTTGGAATCTGAAAGGTGTGGGGAAAGACTGGGTCAAAACT        840
6g20480         ----------------------------------GTGTGGGGAAAGACTGGGTCAAAACT        527
                                                  **************************

GBSS1           TTATGGTCCTAAAACTGGAATTGATTATAGAGATAATCAACTTAGATTCAGCTTGTTGTG        900
6g20480         TTATGGTCCTAAAACTGGAATTGATTATAGAGATAATCAACTTAGATTCAGCTTGTTGTG        584
                ************************************************************
                        Intron 4
GBSS1           TCAGGTAGCTTTTTGCTTTATATACTTTCTACCTATGTTGTTTTACCCTGTATTAATTGT        960
6g20480         TCAG--------------------------------------------------------        584
                ****

GBSS1           TAGATCTGTTGGCATAAACAACTTAATTAAGTGCTTATATGTATTTCTATATTAATAGAA        1020
6g20480         ------------------------------------------------------------        584


GBSS1           GTAAGCAAGTCAAATTGTTTTTCATATAAGTTATAAATTATTTTCATAAGTCATTCTAAA        1080
6g20480         ------------------------------------------------------------        584


GBSS1           GAGTTTTGTAGAAATAAGTTGAAAATTGCTTATAAACATGTCATAAGCCCTTTCTAGAAA        1140
6g20480         ------------------------------------------------------------        584


GBSS1           CCATATTTGCCCTTGACTTATGATTATTGACTACATTTATATGTTGAAATTGGTCTTCAC        1200
6g20480         ------------------------------------------------------------        584


GBSS1           TCTTGACTTTAATGGTTGTTTCCTGTGCTTATTTTCAATCGATAGCTGAAAATTTCAACT        1260
6g20480         ------------------------------------------------------------        584

                                                      Exon 5
GBSS1           TCTTTATATTATTGACTCTAGATATGATCATTATCAATGTGCTTAACTGCTTTCAGGCTG        1320
6g20480         --------------------------------------------------------GCTG        595
                                                                        ****

GBSS1           CACTTGAGGCACCAAGGGTTTTGAACTTAAACAGCAGCAAATATTTCTCAGGACCATATG        1380
6g20480         CACTTGAGGCACCAAGGGTTTTGAACTTAAACAGCAGCAAATATTTCTCAGGACCATATG        655
                ************************************************************
                Intron 5
GBSS1           GTGTGGCTCTCTAGTCTTTACTTTTTTGGTCTTTAATATATTCTTTTGAGGATAAACTTC        1440
6g20480         ------------------------------------------------------------        655
```

```
                                                             Exon 6
GBSS1            AACTTAATTTTACAAGCTTTTTATGACTTTCTACAGGCGAAGATGTAATCTTTGTTGCCA       1500
6g20480          ------------------------------------GCGAAGATGTAATCTTTGTTGCCA       679
                                                     ************************

GBSS1            ATGATTGGCACTCTGCTCTTATTCCATGCTACTTGAAATCGATGTACAAGTCTAGAGGGC       1560
6g20480          ATGATTGGCACTCTGCTCTTATTCCATGCTACTTGAAATCGATGTACAAGTCTAGAGGGC       739
                 ************************************************************
                                  Intron 6
GBSS1            TTTACAAAAACGCAAAGGTGAGGATTTGTTTACTTTTCGTGTTTGCGAAATCTATATAGC       1620
6g20480          TTTACAAAAACGCAAAG-------------------------------------------       756
                 *****************
                                                lam-b Exon 7
GBSS1            ATAAGGAAGCTGACTTTCTTTGTAATCATGACAGGTGGCCTTTTGTATTCATAACATAGC       1680
6g20480          ----------------------------------GTGGCCTTTTGTATTCATAACATAGC       782
                                                   **************************

GBSS1            TTACCAGGGTAGAAACGCCTTTTCAGACTTCTCTCTTCTCAATTTACCTGATGAATTCCG       1740
6g20480          TTACCAGGGTAGAAACGCCTTTTCAGACTTCTCTCTTCTCAATTTACCTGATGAATTCCG       842
                 ************************************************************
                                           Intron 7
GBSS1            AAGCTCTTTCGACTTTATTGATGGGTATGATATGGTTATCCTTCTAAAAATCACATTATA       1800
6g20480          AAGCTCTTTCGACTTTATTGATGG------------------------------------       868
                 ************************
                                                                        Exon 8
GBSS1            ACATCGGTCTTGTGTTCCTACAATTTGATTATGTTTTTCGTTTGTTTCTTTTTCAGGTAT       1860
6g20480          --------------------------------------------------------GTAT       870
                                                                         ****

GBSS1            AATAAGCCTGTGAAGGGAAGAAAAATCAATTGGATGAAGGCTGGAATATTAGAATCCGAC       1920
6g20480          AATAAGCCTGTGAAGGGAAGAAAAATCAATTGGATGAAGGCTGGAATATTAGAATCCGAC       930
                 ************************************************************

GBSS1            CAAGTGTTCACTGTAAGTCCACATTATGCCAAGGAACTTATTTCCGGAGAAGACAGAGGT       1980
6g20480          CAAGTGTTCACTGTAAGTCCACATTATGCCAAGGAACTTATTTCCGGAGAAGACAGAGGT       990
                 ************************************************************

GBSS1            GTGGAATTGGACAATATCATTCGTTCAACGGGCATCATTGGTATCGTTAATGGCATGGAT       2040
6g20480          GTGGAATTGGACAATATCATTCGTTCAACGGGCATCATTGGTATCGTTAATGGCATGGAT       1050
                 ************************************************************

GBSS1            AATCGAGAGTGGAGTCCACAAACTGATAGATACATAGATGTACATTACAATGAAACAACT       2100
6g20480          AATCGAGAGTGGAGTCCACAAACTGATAGATACATAGATGTACATTACAATGAAACAACT       1110
                 ************************************************************
                 Intron 8
GBSS1            GTAAGTAGCACATTTTCAGTTTAACATGGATTCCTTTTATGACATGGAACATAGAAACTG       2160
6g20480          ------------------------------------------------------------       1110

                                                 Exon 9
GBSS1            ATGTTCATCAAAACTGTCATTTTTAGGTCACCGAAGCAAAACCTCTGTTGAAAGAAACTC       2220
6g20480          --------------------------GTCACCGAAGCAAAACCTCTGTTGAAAGAAACTC       1144
                                           **********************************

GBSS1            TTCAAGCCGAGATTGGGTTGCCAGTTGACAGCAGTATCCCTTTGATAGGGTTCATTGGCA       2280
6g20480          TTCAAGCCGAGATTGGGTTGCCAGTTGACAGCAGTATCCCTTTGATAGGGTTCATTGGCA       1204
                 ************************************************************

GBSS1            GGCTAGAAGAGCAGAAAGGTTCAGATATTCTCGTTGAAGCTATTGCCAAGTTCGCTGACG       2340
6g20480          GGCTAGAAGAGCAGAAAGGTTCAGATATTCTCGTTGAAGCTATTGCCAAGTTCGCTGACG       1264
                 ************************************************************
                                       Intron 9
GBSS1            AAAATGTTCAGATAGTAGTTCTTGTAAGTGTCTTTATTAGAATTTTAGTTTCCTTTTTAA       2400
6g20480          AAAATGTTCAGATAGTAGTTCTT-------------------------------------       1286
                 ***********************
                                                                       Exon 10
GBSS1            TGATACTGTTACCTAAACAAAGCTATGTTTATAATTCATATTCTTTTTTACTTCAGGGAA       2460
6g20480          --------------------------------------------------------GGAA       1291
                                                                         ****

GBSS1            CTGGCAAAAAGATCATGGAAAAGCAAATCGAGGTACTGGAGGAAAAATATCCCGGCAAAG       2520
```

```
6g20480        CTGGCAAAAAGATCATGGAAAAGCAAATCGAGGTACTGGAGGAAAAATATCCCGGCAAAG        1351
               ************************************************************

GBSS1          CAATTGGGATAACAAAATTCAACAGCCCCTTGGCTCACAAAATTATTGCTGGAGCTGACT        2580
6g20480        CAATTGGGATAACAAAATTCAACAGCCCCTTGGCTCACAAAATTATTGCTGGAGCTGACT        1411
               ************************************************************

GBSS1          TTATAGTGATCCCAAGTAGATTTGAGCCCTGTGGTCTAGTTCAGTTGCATGCCATGCCCT        2640
6g20480        TTATAGTGATCCCAAGTAGATTTGAGCCCTGTGGTCTAGTTCAGTTGCATGCCATGCCCT        1471
               ************************************************************
                       Intron 10
GBSS1          ATGGAACGGTAGAGTTTATGCGCCTTTTTTCTCCATTTAAGATTTGGATTTTCTATGTTC        2700
6g20480        ATGGAACG----------------------------------------------------        1479
               ********

GBSS1          ATGCAATCACATAGTTGTTGATCAGCATTGTCCGGTCAAAATCGGATGGCTAACATAAAA        2760
6g20480        ------------------------------------------------------------        1479

                              Exon 11
GBSS1          TGCTTGAAACAGGTGCCTATAGTTTCCTCAACTGGTGGACTTGTTGACACTGTGAAAGAA        2820
6g20480        ------------GTGCCTATAGTTTCCTCAACTGGTGGACTTGTTGACACTGTGAAAGAA        1527
                           ************************************************
                                                     Intron 11
GBSS1          GGCTATACAGGATTCCACGCTGGGCCGTTCGATGTAGAGGTAAGAAGATAAGTATCCTTT        2880
6g20480        GGCTATACAGGATTCCACGCTGGGCCGTTCGATGTAGAG---------------------        1566
               ***************************************

GBSS1          TTTTCGTTTTCATTTTGATTGGGAGTGAAACAAAAAGATTATAACTTAAAGTTGTTTTTT        2940
6g20480        ------------------------------------------------------------        1566

                       Exon 12
GBSS1          AGTGTGAAGATGTTGATCCAGATGATGTAGATAAGTTAGCAGCAACTGTAAAGAGAGCCC        3000
6g20480        --TGTGAAGATGTTGATCCAGATGATGTAGATAAGTTAGCAGCAACTGTAAAGAGAGCCC        1624
                 **********************************************************

GBSS1          TTAAAACCTATGGTACTCAAGCCATGAAAGAGATAATCCTGAACTGCATGGCCCAAAACT        3060
6g20480        TTAAAACCTATGGTACTCAAGCCATGAAAGAGATAATCCTGAACTGCATGGCCCAAAACT        1684
               ************************************************************
                              Intron 12
GBSS1          TCTCGTGGAAGGTAAGCGTGTGATTGCCAATTTTGTAGAAATGACATTCAACTAATCCTT        3120
6g20480        TCTCGTGGAAG-------------------------------------------------        1694
               ***********
                                      Exon 13
GBSS1          AATGTGTGATTTCTGATACAGAAACCAGCCAAACTGTGGGAGAAGGCGCTGTTGAACCTA        3180
6g20480        ---------------------AAACCAGCCAAACTGTGGGAGAAGGCGCTGTTGAACCTA        1734
                                    ***************************************

GBSS1          GAAGTCACAGGCAACGTAGCTGGAATAGATGGGGACGAGATTGCTCCTCTTGCAAAGGAA        3240
6g20480        GAAGTCACAGGCAACGTAGCTGGAATAGATGGGGACGAGATTGCTCCTCTTGCAAAGGAA        1794
               ************************************************************

GBSS1          AATGTGGCTACTCCTTGA     3258
6g20480        AATGTGGCTACTCCTTGA     1812
               ******************
```

Mutant	F primer for screening	R primer for screening
lam-a	CGGTGTGTCATCAGCTACTATCTAT	GCACTTAATTAAGTTGTTTATGCCAAC
lam-b	GCTTATAAACATGTCATAAGCCCTTTC	CCCATCAATAAAGTCGAAAGAGCTTC
lam-c	CGGTGTGTCATCAGCTACTATCTAT	GCACTTAATTAAGTTGTTTATGCCAAC

Legend to Supplementary Figure S1

Alignment of genomic and cDNA sequences for starch biosynthetic genes at five genetic loci (A, *rb*; B, *rug3*; C, *rug4*; D, *rug5* and E, *lam*), based on the publicly available whole genome sequence assembly for the pea accession, JI2822, and its associated RNA-Seq resource [31]. The chromosome

position is given for every gene (chr in header of each alignment) and the transcript identity is in the left margin of the alignment in every case. Asterisks underneath the alignments indicate nucleotide identity. The nucleotide change in the mutant allele is indicated in every case (bold red font, grey highlight); for the *rb* mutation, the position of the deletion is indicated, with the flanking two bases in red font. (Note that JI2822 has the genotype *rbrb*). The sequences of the relevant primers are given in the table beneath every gene-cDNA alignment; the positions of forward and reverse primers covering every mutation are indicated in the sequence alignments, highlighted in yellow and blue, respectively. The initiator methionine codon (ATG) is shown in bold for every gene; upstream gene sequence for *lam* is provided (minus numbering). In B, note the non-canonical exon-intron boundary sequence for intron 6 (GC-AG) in *pgm*.
