# Supplementary material for: A Suite of Pea (Pisum sativum L.) Near-Isolines: Genetic Resources and Molecular Tools to Breed for Seed Carbohydrate and Protein Quality in Legumes
Source: Int J Mol Sci. 2025 Mar 14;26(6):2612. doi: 10.3390/ijms26062612 (PMC11942445; doi:10.3390/ijms26062612)
Supplement: Supplementary file 1 [file ijms-26-02612-s001.zip › ijms-3410026-Supplementary Figures S2-S6.pdf]

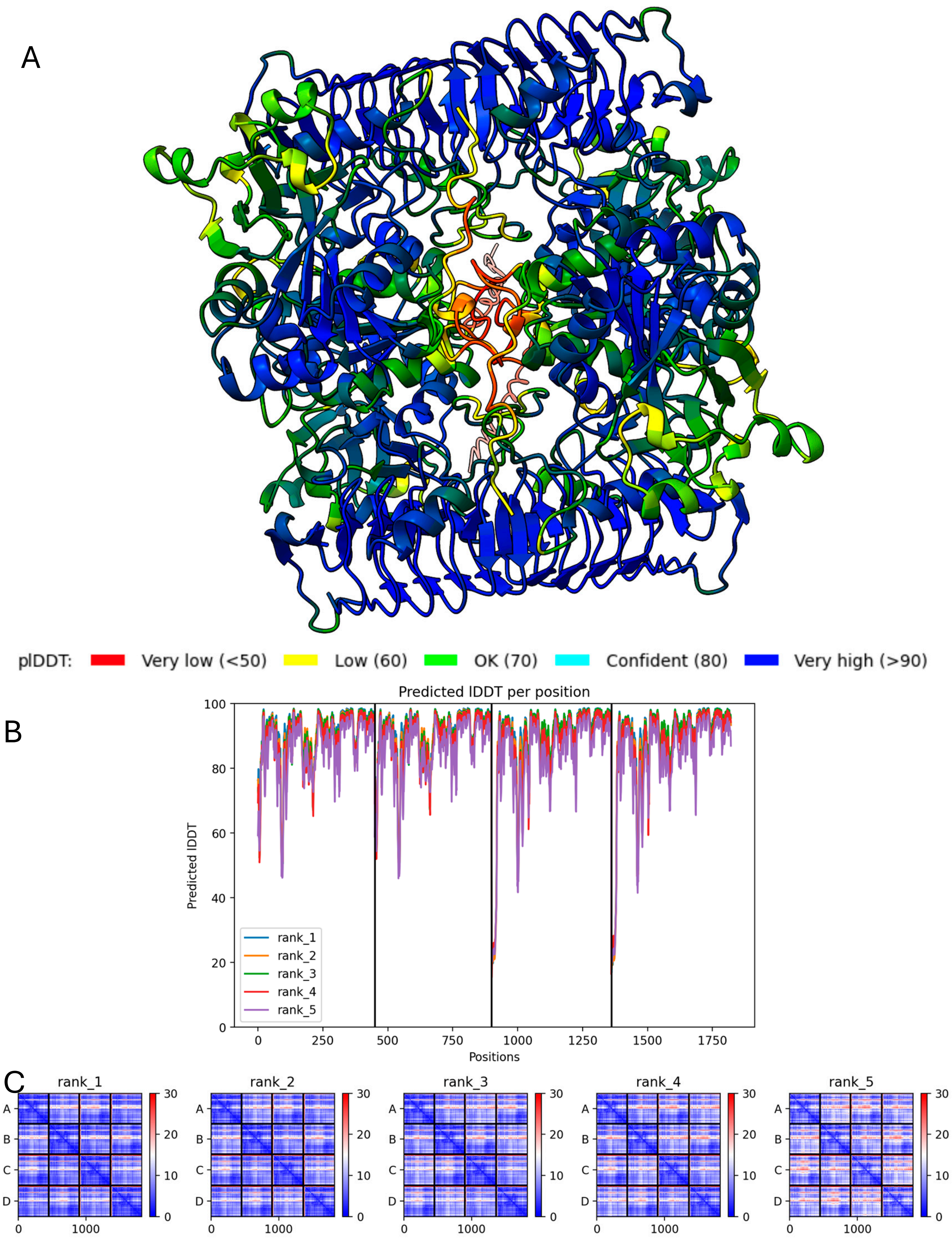

Supplementary Figure S2. AGPS1, AGPS2, AGPL1, AGPL1 tetramer. A: Model coloured by PIDDT score to show level of confidence in predicted model. B: Graph of PIDDT score. C: PAE plots to indicate relationships between pairs of residues in the structure prediction and the confidence in their positions. Referenced related PDB accession code: 1YP4

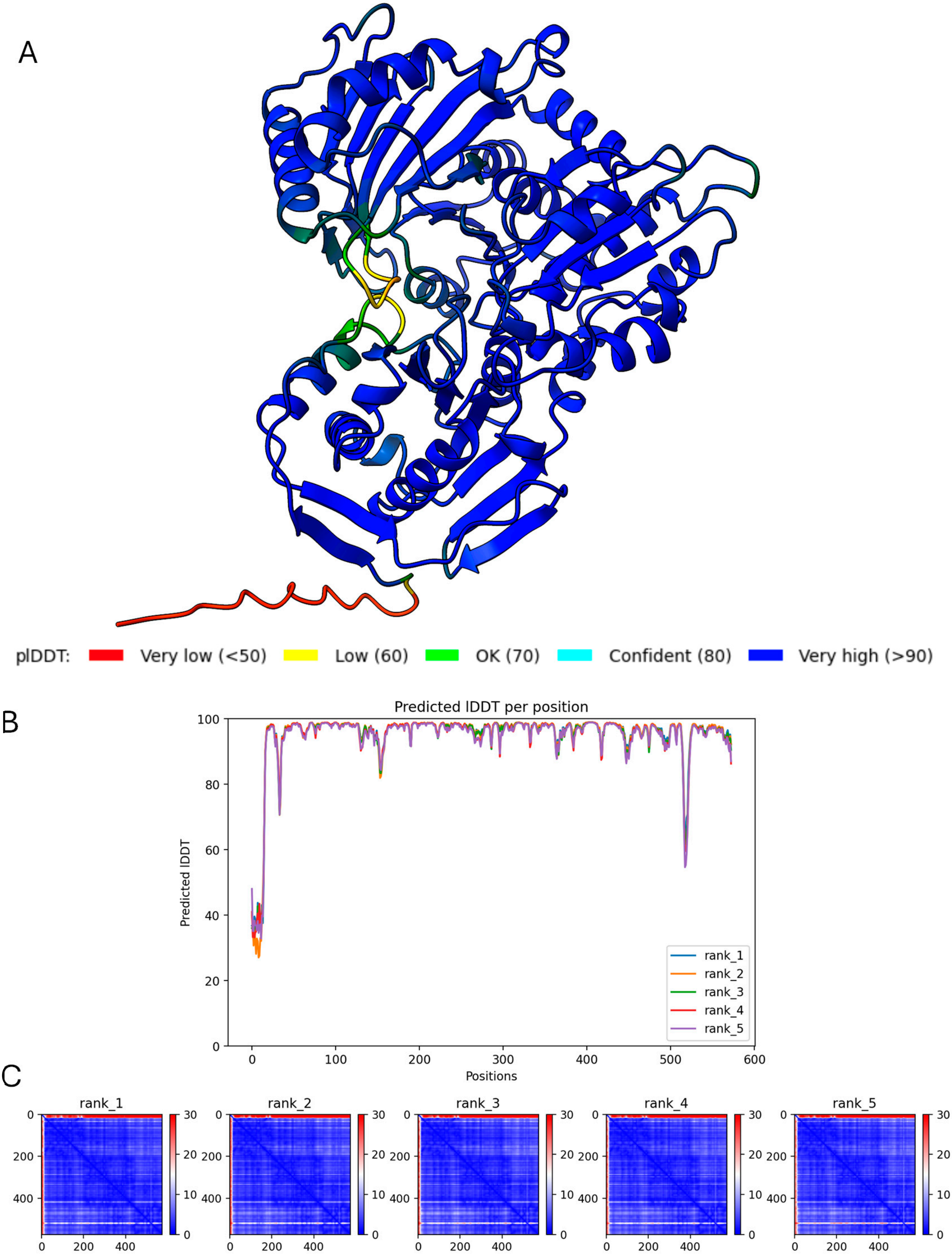

Supplementary Figure S3. pPGM. A: Model coloured by PlDDT score to show level of confidence in predicted model. B: Graph of PlDDT score. C: PAE plots to indicate relationships between pairs of residues in the structure prediction and the confidence in their positions. Referenced related PDB accession code: 6SNO.

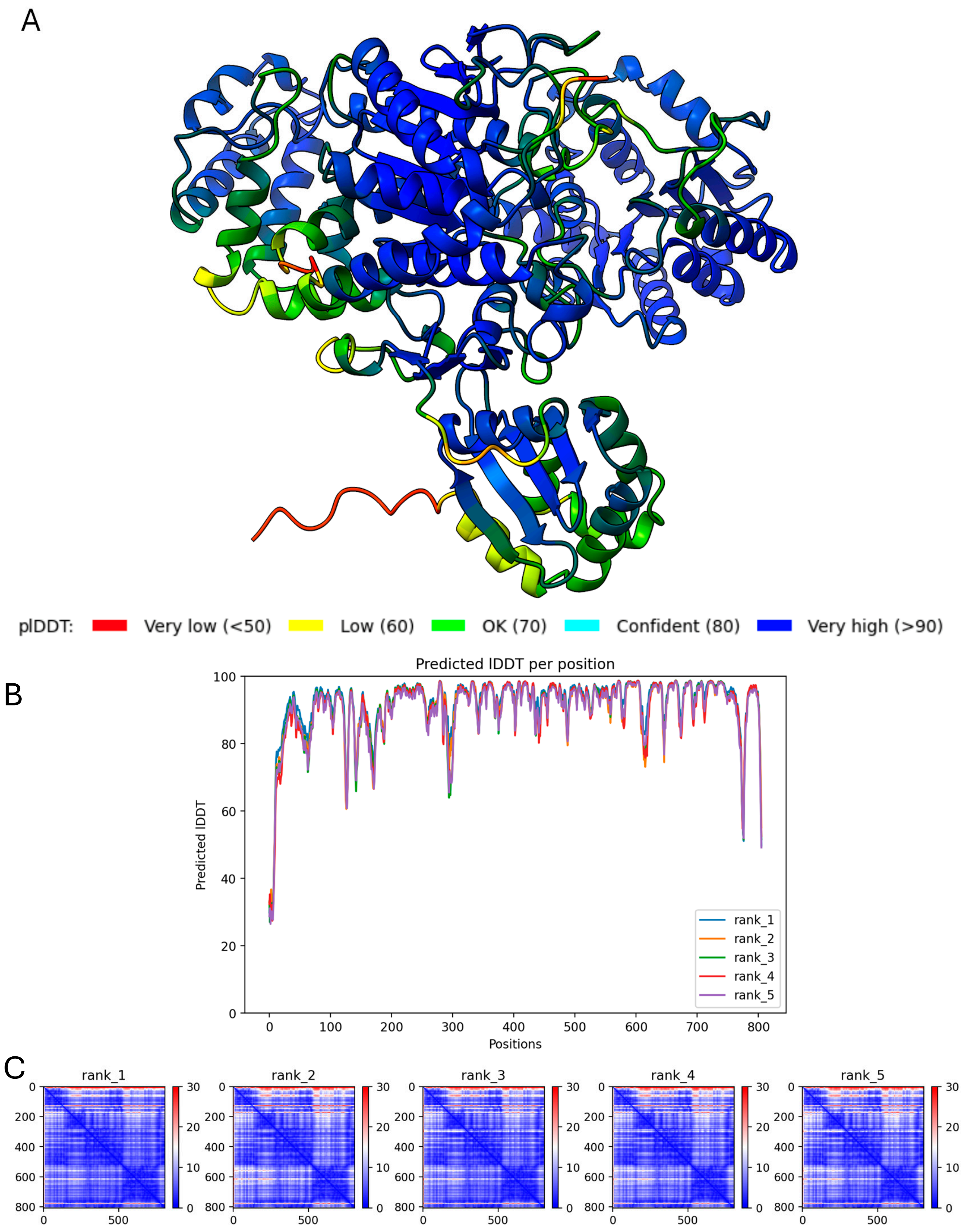

Supplementary Figure S4. SUS1. A: Model coloured by PlDDT score to show level of confidence in predicted model. B: Graph of PlDDT score. C: PAE plots to indicate relationships between pairs of residues in the structure prediction and the confidence in their positions. Referenced related PDB accession code: 3S27

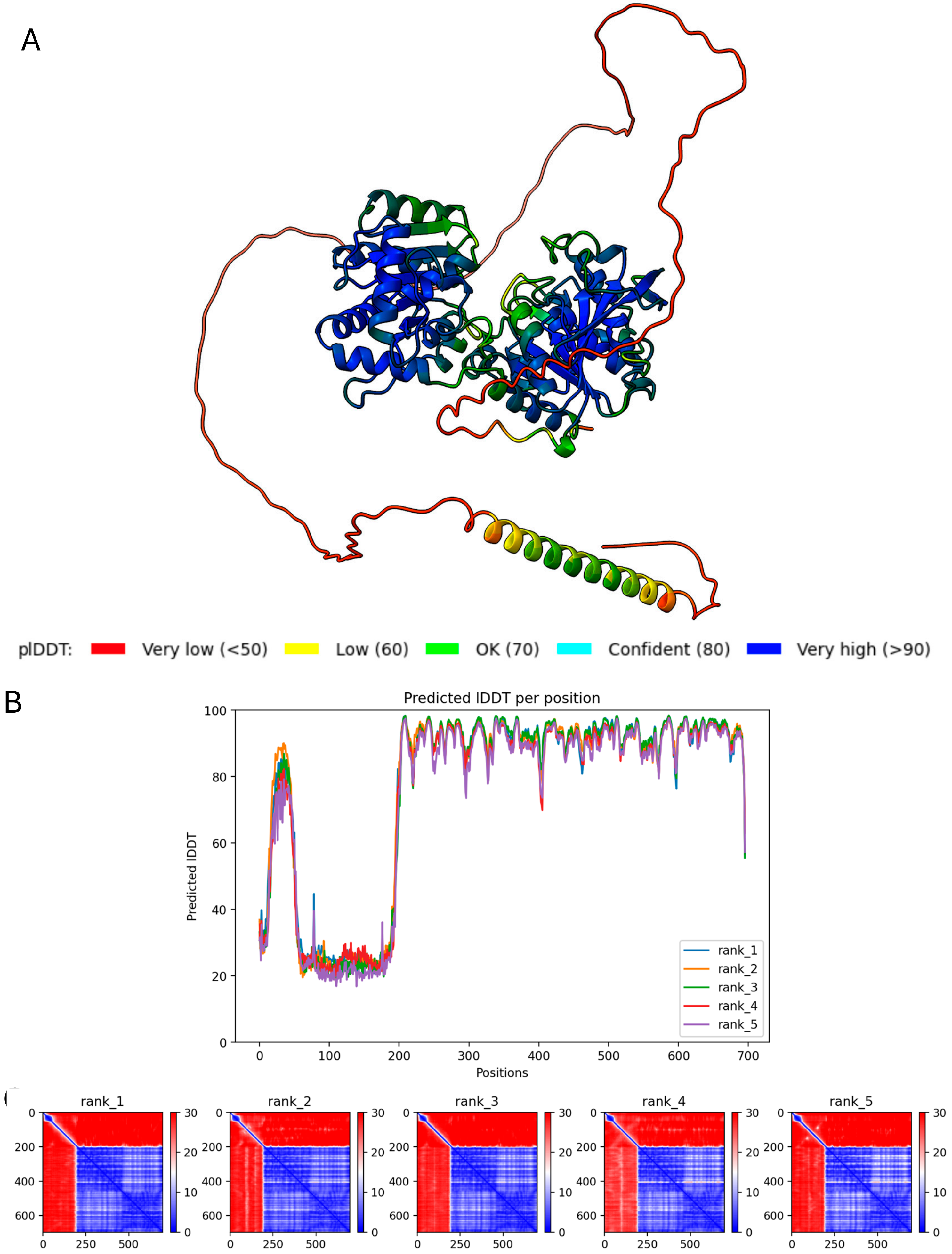

Supplementary Figure S5. SS2. A: Model coloured by PlDDT score to show level of confidence in predicted model. B: Graph of PlDDT score. C: PAE plots to indicate relationships between pairs of residues in the structure prediction and the confidence in their positions. Referenced related PDB accession codes: 4HLN, 6GNF.

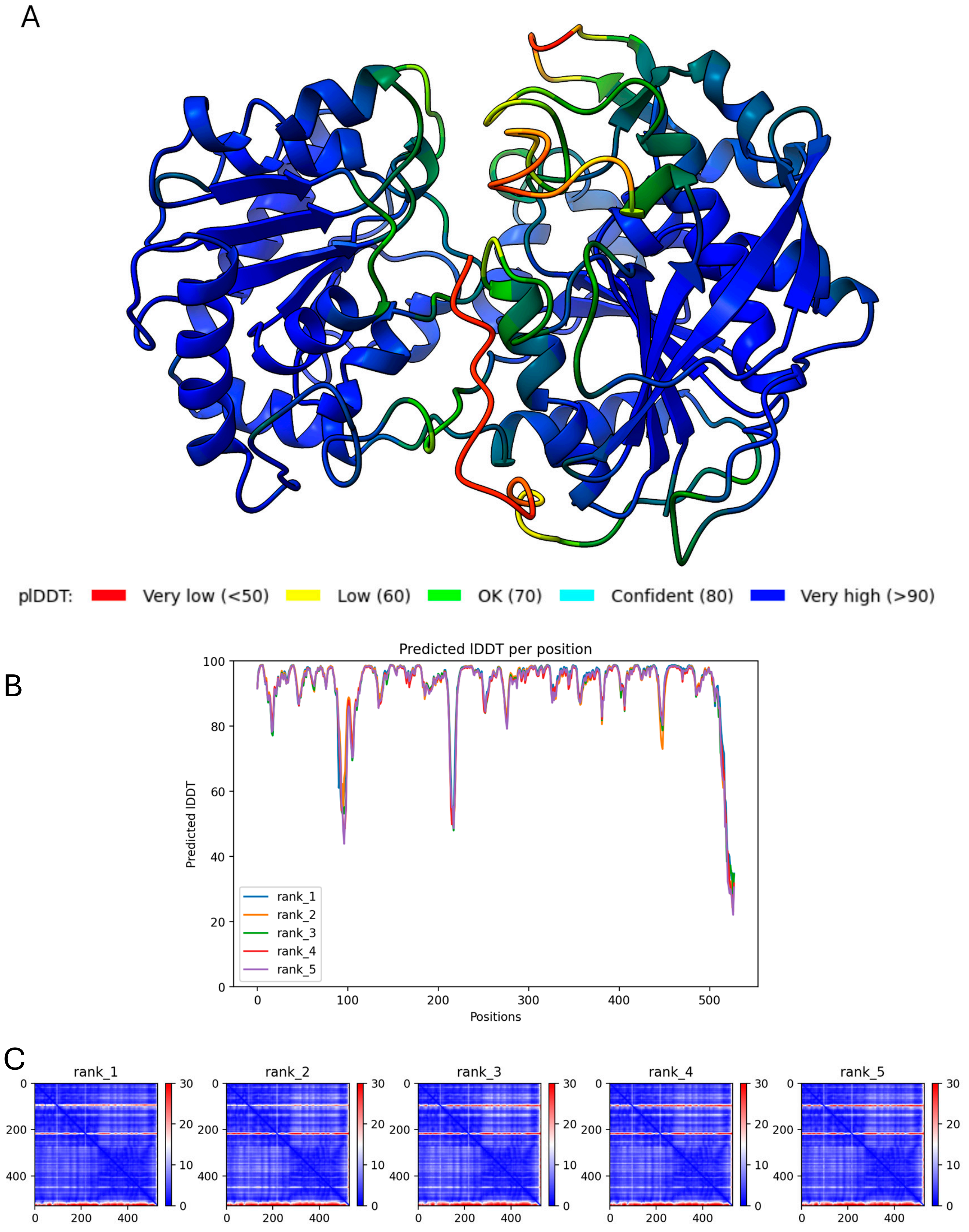

Supplementary Figure S6. GBSS1. A: Model coloured by PIDDT score to show level of confidence in predicted model. B: Graph of PIDDT score. C: PAE plots to indicate relationships between pairs of residues in the structure prediction and the confidence in their positions. Referenced related PDB accession codes: 3CX4, 3VUF
