# Supplementary material for: A Suite of Pea (Pisum sativum L.) Near-Isolines: Genetic Resources and Molecular Tools to Breed for Seed Carbohydrate and Protein Quality in Legumes
Source: Int J Mol Sci. 2025 Mar 14;26(6):2612. doi: 10.3390/ijms26062612 (PMC11942445; doi:10.3390/ijms26062612)
Supplement: Supplementary file 1 [file ijms-26-02612-s001.zip › ijms-3410026-Supplementary Table S1.pdf]

Supplementary Table S1. Summary of mutations and associated amplicon sizes for alleles of six starch biosynthetic genes in pea

Mutant	Genomic change (cDNA)	Genomic Change (gDNA)	F primer	R primer	Product
r	A 2577-insertion element*	A 27379-(insertion)-G2780*	CCTTGCGGATGGAGTTGAA	AACTGACAGCTCAATTGG	109 bp cDNA; 109 bp gDNA
r-c	G 1712 A	G 22984 A	ATTCACGGGCTCTTCCC	GAAGCTCAATCCACTTGTCTGC	141 bp cDNA; 262 bp gDNA
r-d	G 1484 A	G 20772 A	GGCCTGAACATGTTTGATGG	CTGCAGTCCATGATGAGTGTAC	216 bp cDNA; 319 bp gDNA
r-f	G 2052 A	G 25551 A	TTTATGGGGAATGAATTCGG	CCATGATATCTTAGATAATCTGCATCAC	158 bp cDNA; 696 bp gDNA
r-g	C 2134 T	G 25633 A	TTTATGGGGAATGAATTCGG	CCATGATATCTTAGATAATCTGCATCAC	158 bp cDNA; 696 bp gDNA
r-h	n.a.	G 24153 A	GGCTGTTGCAGACAAGTGG	GTCAGTGTGTGGACTATATCACCC	81 bp cDNA; 1244 bp gDNA
r-i	n.a.	G 13242 A	TCTTCTTGCCAAACAATGCC	GTTCTTGGATAGCCATAATCTGG	360 bp cDNA; 8433 bp gDNA
r-j	G 1819 A	G 24218 A	AAGATGAAGATTGGAGAATGGG	AGTAGATGGCCTGTCTAAAGCC	179 bp cDNA; 1179 bp gDNA
r-k	n.a.	G 24299 A	ATGACCAGGCCTTGGTTG	AGTAGATGGCCTGTCTAAAGCC	92 bp cDNA; 1092 bp gDNA

Mutant	Genomic change (cDNA)	Genomic change (gDNA)	F primer	R primer	Product
*rb***	9 bp deletion**	T 470-G471 (9 bp deletion)**	(1) GCAAGTGCCTTCATTTCTGAG	(5) GCATCCTCCAACAGGAACC	236 bp (using F1 and R5); 354 bp (F1 and R8)
			(5) CCTCTTACCAAACGAGCTGC	(8) CGAAATAGGTGCGAGCG	266 bp (using F5 and R8)
rb-d	G 1367 A	G 2802 A	GAATCCGAAATCGCTTCCC	CGATGACAACTTCTTTCCCG	121 bp cDNA; 207 bp gDNA
rb-f	G 912 A	G 1924 A	GCAAGTAGATACTTCTCGTCTTGG	ACATTGTGCTCTCTTATTGCGG	183 bp cDNA; 183 bp gDNA
rb-h	G 520 A	G 1153 A	TGGAGATGGATTTGTGGAGG	TGAACACATAAACTCCCATAGATGC	431 bp cDNA; 1158 bp gDNA

Mutant	Genomic change (cDNA)	Genomic change (gDNA)	F primer	R primer	Product
rug3-a	G 383 A	G 703 A	CAGACTCGACAACTTCATCATCTC	AGGGTGTTTCCGTAAATCTTG	642 bp cDNA; 1905 bp gDNA
rug3-b	C 1699 T	C 4367 T	TACGTCCTCCAGTTTGCC	AAGGCAATTTGAGCATCG	212 bp cDNA; 402 bp gDNA
rug3-c	G 583 A	G 1479 A	TATTGTCAACGCCAGCC	AGGGTGTTTCCGTAAATCTTG	178 bp cDNA; 629 bp gDNA
rug3-d	C 391 T	C 711 T	CAGACTCGACAACTTCATCATCTC	AGGGTGTTTCCGTAAATCTTG	642 bp cDNA; 1905 bp gDNA
rug3-e	CT deletion 1430/1431	CT deletion 3757/3758	GATGCTGGAAATCTGTCG	ATCTTATTCGCGCCTTCAG	251 bp cDNA; 430 bp gDNA

Mutant	Genomic change (cDNA)	Genomic change (gDNA)	F primer	R primer	Product
rug4-a	C 490 T	C 788 T	TGGTTGAAAATTTGCAACC	TAGCTGTGAAGTCTGAGAAATTCC	244 bp cDNA; 332 bp gDNA
rug4-b	G 1733 A	G 2668 A	TTACACCGAAACTAGCCGC	ATTCAACTTGTAGGTCTCGATATGTTC	313 bp cDNA; 408 bp gDNA
rug4-c	n.a.	G 1966 A	CTTGAGACTTTCCTTGACAGAATCCC	CAGGATACTTAGTCTTCTCAAGTGC	918 bp gDNA

Mutant	Genomic change (cDNA)	Genomic change (gDNA)	F primer	R primer	Product
rug5-a	G 2124 A	G 3702 A	GGAACCGGAAGAGCTGATTT	GAAAGCCCTTCGTTTTGATCA	507 bp cDNA; 507 bp gDNA
rug5-b	G 841 A	G 1926 A	ACAAGCTGTACGAGAGACCT	CGTACTCCTATATCGTGTGCTTC	343 bp cDNA; 667 bp gDNA
rug5-c	G 2075 A	G 3653 A	GGAACCGGAAGAGCTGATTT	GAAAGCCCTTCGTTTTGATCA	507 bp cDNA; 507 bp gDNA

Mutant	Genomic change (cDNA)	Genomic change (gDNA)	F primer	R primer	Product
lam-a	G 253 A	G 253 A	CGGTGTGTCATCAGCTACTATCTAT	GCACTTAATTAAGTTGTTTATGCCAAC	1153 bp gDNA
lam-b	n.a.	G 1654 A	GCTTATAAACATGTCATAAGCCCTTTC	CCCATCAATAAAGTCGAAAGAGCTTC	658 bp gDNA
lam-c	n.a.	G 454 A	CGGTGTGTCATCAGCTACTATCTAT	GCACTTAATTAAGTTGTTTATGCCAAC	1153 bp gDNA

Sequence coordinates for every mutation are based on the genome sequence of JI2822 and associated RNA-Seq data [31]. Note that JI2822 has the genotype *rbrb* **, having a nine base pair exonic deletion. Sequences of forward and reverse primers used for screening and sequencing are listed. The positions of mutations are indicated for both genomic and cDNA, where primers are relevant to the latter; not applicable (n.a.) indicates where the mutation is intronic. The *rb*, *rug3-b* , *rug4-b* and *rug5-b* mutants were described previously [38, 20, 22, 24, respectively]. Analysis of *r* mutants was provided in earlier work [30]. * an insertion element of ~1 kb in *r* [38].
